# Induction of Fungal Secondary Metabolites by Co-Culture with Actinomycete Producing HDAC Inhibitor Trichostatins

**DOI:** 10.4014/jmb.2301.01017

**Published:** 2023-07-26

**Authors:** Gwi Ja Hwang, Jongtae Roh, Sangkeun Son, Byeongsan Lee, Jun-Pil Jang, Jae-Seoun Hur, Young-Soo Hong, Jong Seog Ahn, Sung-Kyun Ko, Jae-Hyuk Jang

**Affiliations:** 1 Chemical Biology Research Center, Korea Research Institute of Bioscience and Biotechnology (KRIBB), Cheongju 28116, Republic of Korea; 2 Department of Biomolecular Science, KRIBB school of Bioscience, University of Science and Technology (UST), Daejeon 34141, Republic of Korea; 3 Korean Lichen Research Institute, Sunchon National University, Suncheon 57922, Republic of Korea; 4 Antimicrobial Discovery Center, Department of Biology, Northeastern University, Boston 02115 MA, USA

**Keywords:** Co-culture system, fungal cryptic secondary metabolites, structure determination, physical interaction

## Abstract

A recently bioinformatic analysis of genomic sequences of fungi indicated that fungi are able to produce more secondary metabolites than expected. Despite their potency, many biosynthetic pathways are silent in the absence of specific culture conditions or chemical cues. To access cryptic metabolism, 108 fungal strains isolated from various sites were cultured with or without *Streptomyces* sp. 13F051 which mainly produces trichostatin analogues, followed by comparison of metabolic profiles using LC-MS. Among the 108 fungal strains, 14 produced secondary metabolites that were not recognized or were scarcely produced in mono-cultivation. Of these two fungal strains, *Myrmecridium schulzeri* 15F098 and *Scleroconidioma sphagnicola* 15S058 produced four new compounds (1-4) along with a known compound (5), demonstrating that all four compounds were produced by physical interaction with *Streptomyces* sp. 13F051. Bioactivity evaluation indicated that compounds **3-5** impede migration of MDA-MB-231 breast cancer cells.

## Introduction

Fungi are major composers of ecosystems and producer of new secondary metabolites that are used in medicine and agriculture [1]. To date, 100,000 fungi have been identified and approximately 5 million fungal species may exist [2]. A recent whole-genome sequencing analysis indicated that fungi have the potential to produce more secondary metabolites than expected [3]. Despite this genetic capability, many biosynthetic pathways are silent or the rediscovery of secondary metabolites is frequent under traditional laboratory culture conditions [4, 5]. Various strategies have been developed to access cryptic metabolites [6-8]. Co-culture of two microorganisms in the same culture environment is a representative strategy [9, 10]. This strategy induces physical and metabolite interactions between the two microorganisms, which ultimately increases gene expression, and consequently increases metabolite production. Another approach is epigenetic manipulation at the chromatin level using histone deacetylase (HDAC) and DNA methyltransferase (DNMT) inhibitors, which regulate transcriptional gene expression in fungi [11, 12].

In a previous study, we examined the chemical profiles of actinomycetes isolated from soil samples collected from Ulleung Island by liquid chromatography-photodiode array-mass spectrometry (LC-PDA-MS) [13]. Among the 208 actinomycete strains isolated, *Streptomyces* sp. 13F051 produced trichostatin analogues. Trichostatin A (TSA) is a HDAC inhibitor, its mechanism of action has been established; the hydroxamate group of TSA chelates a zinc ion in the active site pocket of HDAC, blocking the catalytic reaction [14]. Owing to its potent HDAC inhibitory activity, TSA has been used to enhance the cryptic secondary metabolites of fungi [15].

We hypothesized that if fungi were cultured with an actinomycete, producing HDAC inhibitors, TSA analogues, novel fungal secondary metabolites are induced by HDAC inhibitors or physical interaction between two microorganisms. To test this hypothesis, various fungal strains were co-cultured with an actinomycete on agar plates, and their metabolic profiles were monitored by LC-MS. The co-culture of *Streptomyces* sp. 13F051 and the three fungal strains produced secondary metabolites that were not recognized or were scarcely produced in mono-cultures. To determine whether the induced metabolites were produced by HDAC inhibitors or physical interactions, the four fungal strains were cultured with a disk containing acetone extracts of *Streptomyces* sp. 13F051. The two fungal strains produced the novel secondary metabolites via physical interaction in the co-culture system. Here we report four new fungal secondary metabolites: dinapinone analogues (**1** and **2**), sambutoxin analogues (**3** and **4**) and a known compound AS2077715 (**5**) [16] induced by physical interaction in the co-culture system. After isolation, all the compounds were further studied to elucidate their structure and biological activities.

## Materials and Methods

### General Experimental Procedures

Optical rotations were measured using a JASCO P-1020 polarimeter (JASCO, Japan). UV spectra were recorded on a NP80 NanoPhotometer (IMPLEN, Germany). Nuclear magnetic resonance (NMR) spectra were obtained in CD_3_OD:CDCl_3_ co-solvent as the internal standards (*δ*_H_ 3.31/*δ*_C_ 49.1) and (*δ*_H_ 7.24/*δ*_C_ 77.2), respectively or DMSO-*d*_6_ (*δ*_H_ 2.49/*δ*_C_ 39.5) using a Bruker AVANCE HD 700 MHz NMR spectrometer (Bruker, Germany) at the Korea Basic Science Institute (KBSI) in Ochang, Korea. High-resolution electrospray ionisation mass spectrometry (HRESIMS) were recorded on a Waters Synapt G2 mass spectrometer (Waters, USA) at the KBSI in Ochang, Korea. Analytical C_18_ (Nacalai Tesque, Japan, 5 μm, 4.6 × 150 mm), analytical C_30_ (Nomura Chemical, Japan, 5 μm, 4.6 × 150 mm) and semipreparative C_18_ (Nacalai Tesque, Japan; 10 μm, 10 × 250 mm) columns were used for reversed-phase HPLC equipped with a Waters 2996 photodiode array detector (Waters). High-performance liquid chromatography (HPLC) grade solvents (Burdick & Jackson, USA) were used. Liquid chromatography-mass spectrometry (LC-MS) analysis was performed using an LTQ XL linear ion trap (Thermo Scientific, USA) equipped with an electrospray ionization (ESI) source coupled with a rapid separation LC (RSLC; ultimate 3000, Thermo Scientific) system (ESI-LC-MS).

### Strain Identification

Soil samples were collected from Ulleung Island, Republic of Korea [13]. Among the isolated strains, 13F051 displayed the most similar 16S rRNA gene sequence (GenBank Accession No. MW774247) to *Streptomyces sioyaensis* NRRL B-5408 (99%, 1442/1449), *Streptomyces inhibens* NEAU-D10 (99%, 1442/1449), and *Streptomyces auratus* NRRL 8097 (99%, 1439/1449). Therefore, strain 13F051 was named *Streptomyces* sp. 13F051 and was used in subsequent culture experiments.

Fungal strain 15F098 was isolated from soil samples collected from Jeju Island. BLAST similarity search analysis indicated that the internal transcribed spacer (ITS) sequence of the 15F098 fungal strain had the most similar ITS sequence (GenBank Accession No. OL376480) to that of *Myrmecridium schulzeri* CSB_F134 (99%, 480/485), *Myrmecridium schulzeri* MS3 (98%, 502/513), and *Myrmecridium schulzeri* PWQ2293 (98%, 491/499). Therefore, the strain 15F098 was identified as *Myrmecridium schulzeri* 15F098.

The endolichenic fungal strain 15S058 was provided by the Korea Lichen & Allied Bioresources Center (KOLABIC), Korea Lichen Research Institute (KoLRI), Sunchon National University [17]. This strain had the most similar ITS sequence (GenBank Accession No. OK639166) to that of *Scleroconidioma sphagnicola* NK08 (99%, 568/569), *Scleroconidioma* sp. K22W31 (99%, 566/567), and *Dothideomycetes* sp. JMUR-2016 (99%, 549/550). Therefore, strain 15S058 was identified as *Scleroconidioma sphagnicola* 15S058 (Institutional code: KoLRI031248).

### Fermentation

*Streptomyces* sp. 13F051 was maintained on a 60-mm petri dish containing soluble starch and yeast extract (SY) agar medium (soluble starch, 10.0 g; yeast extract, 1.0 g; tryptone, 1.0 g; agar, 18.0 g in 1 L of distilled water) at 28°C for 3 days. The two fungal strains were maintained on a 60-mm petri dish in potato dextrose (PD) agar medium (dextrose, 20.0 g; potato extract, 4.0 g; agar, 18.0 g in 1 L of distilled water). The soil derived fungus *M. schulzeri* 15F098 was cultured at 28°C for 7 days. The endolichenic fungus *S. sphagnicola* 15S058 was cultured at 25°C for 7 days.

For each co-culture experiment, *Streptomyces* sp. 13F051 was circularly inoculated with a 10-mm diameter at the center of a 60-mm petri dish using an inoculation loop. Two agar pieces (10 × 10 mm) of each fungal strain were placed on both sides of *Streptomyces* sp. 13F051. The co-culture dishes containing ISP2 medium (yeast extract, 4.0 g; malt extract, 10.0 g; dextrose, 4.0 g; agar, 20.0 g in 1 L of distilled water) of *Streptomyces* sp. 13F051 and *M. schulzeri* 15F098 were incubated at 28°C for 14 days. *Streptomyces* sp. 13F051 and *S. sphagnicola* 15S058 were co-incubated on 60-mm petri dish containing Malt extract (MYE) agar medium (malt extract, 6.0 g; maltose, 1.8 g; dextrose, 6.0 g; yeast extract, 1.2 g; agar, 18 g in 1 L of distilled water) at 25°C for 14 days. Scaled-up cultures were followed the same process.

### Extraction and Isolation

Each cultured agar media (60-mm dish × 100) was soaked with acetone three times, and acetone was removed from the organic solvent fraction by evaporation. A acetone extracts (*Streptomyces* sp. 13F051 with *M. schulzeri* 15F098, 0.8 g; *S. sphagnicola* 15S058, 1.2 g) were fractionated by ODS chromatography with a stepwise solvent system of MeOH-H_2_O (from 20:80, 40:60, 60:40, 80:20, to 100:0; 1 L for each step). Fraction 5 from *M. schulzeri* 15F098 and *Streptomyces* sp. 13F051 co-culture extracts was purified by reversed-phase HPLC (Nacalai Tesque-semipreparative C_18_, 50%–85% CH_3_CN over 40 min, 3 ml/min, UV detection at 270 and 370 nm) to give compounds **1** and **2** (3.3 mg, *t*_R_ 30.1 min). Compound **1** (2.7 mg, *t*_R_ 23.8 min) and **2** (0.3 mg, *t*_R_ 21.5 min) were isolated by reversed-phase HPLC (Nomura chemical-analytic C_30_, 70% CH_3_CN over 50 min, 0.8 ml/min). Compounds **3** (0.9 mg, *t*_R_ 34.5 min), **4** (1.2 mg, *t*_R_ 44.1 min), and **5** (1.8 mg, *t*_R_ 15.5 min) produced by co-culture system of *S. sphagnicola* 15S058 and *Streptomyces* sp. 13F051 were isolated by reversed-phase HPLC (Nacalai Tesque-semipreparative C_18_, 55%–80% CH_3_CN over 50 min, 3 ml/min).

Dinapinone analogue 1 (**1**). Brown amorphous powder;${\left[\text{α}\right]}_{\text{D}}^{\text{25}}$+205.0 (c 0.1, MeOH); UV (MeOH) λ_max_ (log ε) 222 (3.33), 289 (4.75), 384 (3.68) nm; ^1^H and ^13^C NMR data, Table 1; HRESIMS *m/z* 945.4249 [M + Na]^+^ (calcd for C_50_H_66_O_16_Na, 945.4249).

Dinapinone analogue 2 (**2**). Brown amorphous powder;${\left[\text{α}\right]}_{\text{D}}^{\text{25}}$-188.1 (c 0.1, MeOH); UV (MeOH) λ_max_ (log ε) 222 (3.32), 290 (4.75), 383 (3.66) nm; HRESIMS *m/z* 923.4433 [M + H]^+^ (calcd for C_50_H_67_O_16_, 923.4429).

Sambutoxin analogue 1 (**3**). White amorphous powder;${\left[\text{α}\right]}_{\text{D}}^{\text{25}}$-35.92 (c 0.05, MeOH); UV (MeOH) λ_max_ (log ε) 255 (4.02), 295 (3.69) nm; ^1^H and ^13^C NMR data, Table 2; HRESIMS *m/z* 436.2465 [M + Na]^+^ (calcd for C_29_H_34_N_4_O_6_Na, 436.2464).

Sambutoxin analogue 2 (**4**). White amorphous powder;${\left[\text{α}\right]}_{\text{D}}^{\text{25}}$-73.64 (c 0.05, MeOH); UV (MeOH) λ_max_ (log ε) 255 (4.12), 295 (3.69) nm; ^1^H and ^13^C NMR data, Table 2; HRESIMS *m/z* 450.2620 [M + Na]^+^ (calcd for C_29_H_34_N_4_O_6_Na, 450.2620).

### Induction Mechanism Monitoring Assay

*Streptomyces* sp. 13F051 was cultured following the same culture process as for co-culture with each fungal strain. The cultured agar media were soaked in acetone three times and evaporated to remove the solvent. *M. schulzeri* 15F098 was cultured on ISP2 agar media with a paper disk containing the acetone extracts of *Streptomyces* sp. 13F051 (0.5 mg/disk). *S. sphagnicola* 15S058 was cultured with acetone extracts (0.5 mg/disk) of *Streptomyces* sp. 13F051 on MYE agar media. *Streptomyces* sp. KCB13F003 was cultured with two fungal strains and monitored using the same process.

### Antibacterial Assay

Two gram-positive bacteria (*Enterococcus faecalis* KCTC 5191 and *Bacillus subtilis* KCTC 1021) and two gram-negative bacteria (*Escherichia coli* CCARM 1356 and *Pseudomonas aeruginosa* KCTC 2004) were cultured using Mueller-Hinton broth (5 ml/15 ml tube) at 28°C for 24 h. Then, 20 μl of 1 × 10^5^ CFU/ml was inoculated on Brain Heart Infusion (HBI) agar plates (90-mm), followed by loading the disks, which contained 50 μg of mixture (**1** and **2**) and **3-5**. After incubation at 28°C for 72 h, antibacterial activity was evaluated by measuring the diameter of the growth inhibition zone.

### Cell Viability Assay

HeLa (human cervical cancer), MDA-MB-231 (human breast cancer), Neuro-2a (mouse neuroblastoma), and PC12 (pheochromocytoma of rat adrenal medulla) cell lines were maintained in Dulbeccós modified Eaglés medium (LM 001-05, DMEM, Welgene, Korea) containing 10% fetal bovine serum (FBS; Welgene, Korea, S 001-07), 100 units penicillin, and 100 μg/ml streptomycin (15140-122, Gibico, USA) at 37°C with 5% CO_2_ in a humidified atmosphere. Each cell line was seeded into 96-well cell culture plates (1 × 10^4^ cells/well) and incubated for 16 h. The cells were then treated with various concentrations of the test compounds. After incubation for 24 h, 10 μl of EZ-Cytox colorimetric assay (0793, Daeil Lab service, Korea) was directly added and incubated at 37°C for 2 h. Absorbance was measured at 450 nm using a microplate reader (Spectra Max 190, Molecular Devices, USA). Cell viability was normalized to that of control cells.

### Scratch Wound Healing Assay

MDA-MB-231 cells were seeded overnight in 24-well cell culture plates (8 × 10^4^ cells/well). Each well was artificially scratched using a Scar scratcher (201925, SPL, Korea). The cells were incubated in DMEM containing 1 μg/ml mitomycin C for 3 h, followed by incubation with the test compounds at the indicated concentrations. After incubation for 24 h, the cells were fixed in 4% paraformaldehyde for 15 min, stained with 0.2% crystal violet, and imaged under a microscope. Wound closure areas were measured using ImageJ (Software 1.48q, Rayne Rasband, National Institutes of Health, USA)

### Time-lapse Cell Tracking Analysis.

MDA-MB-231 cells (8 × 10^3^ cells/100 μl) were seeded on μ-Slide I (80106, ibidi, Germany). After 4 h, 900 μl DMEM was added to the slide. After incubation for 16 h, cells were treated with indicated concentrations of compound and directly transferred to a HoloMonitor M4 time-lapse cytometer (Phase Holographic Imaging) kept in a 37°C incubator. Live cells were imaged every 15 min for 12 h. Migration was analyzed using the HoloStudio M4 software.

## Results

### Strain Selection using the Co-Culture System

A total of 108 fungal strains were co-cultured with *Streptomyces* sp. 13F051 producing the HDAC inhibitor trichostains, on agar media for 14 days, with the goal of increasing the production of cryptic metabolites. The metabolic profiles of mono- and co-cultured fungi were monitored by LC-MS to detect newly enhanced peaks in the co-culture system. The 14 fungal strains were selected as the candidates to produce the secondary metabolites that were not recognized or scarcely produced in mono-cultures. Finally, three fungal strains that produced novel secondary metabolites were selected for further studies based on searches of public and in-house databases with UV and MS data.

To identify the producer of the induced compounds, we monitored the chemical profiles of each mono-cultured extracts by LC-MS. The fungal strains produced the induced compounds, which was also confirmed by previous studies and are discussed below.

### Induction Factors for Fungal Derived Compounds (1−5) in Co-Culture System

**Fig. 1:**
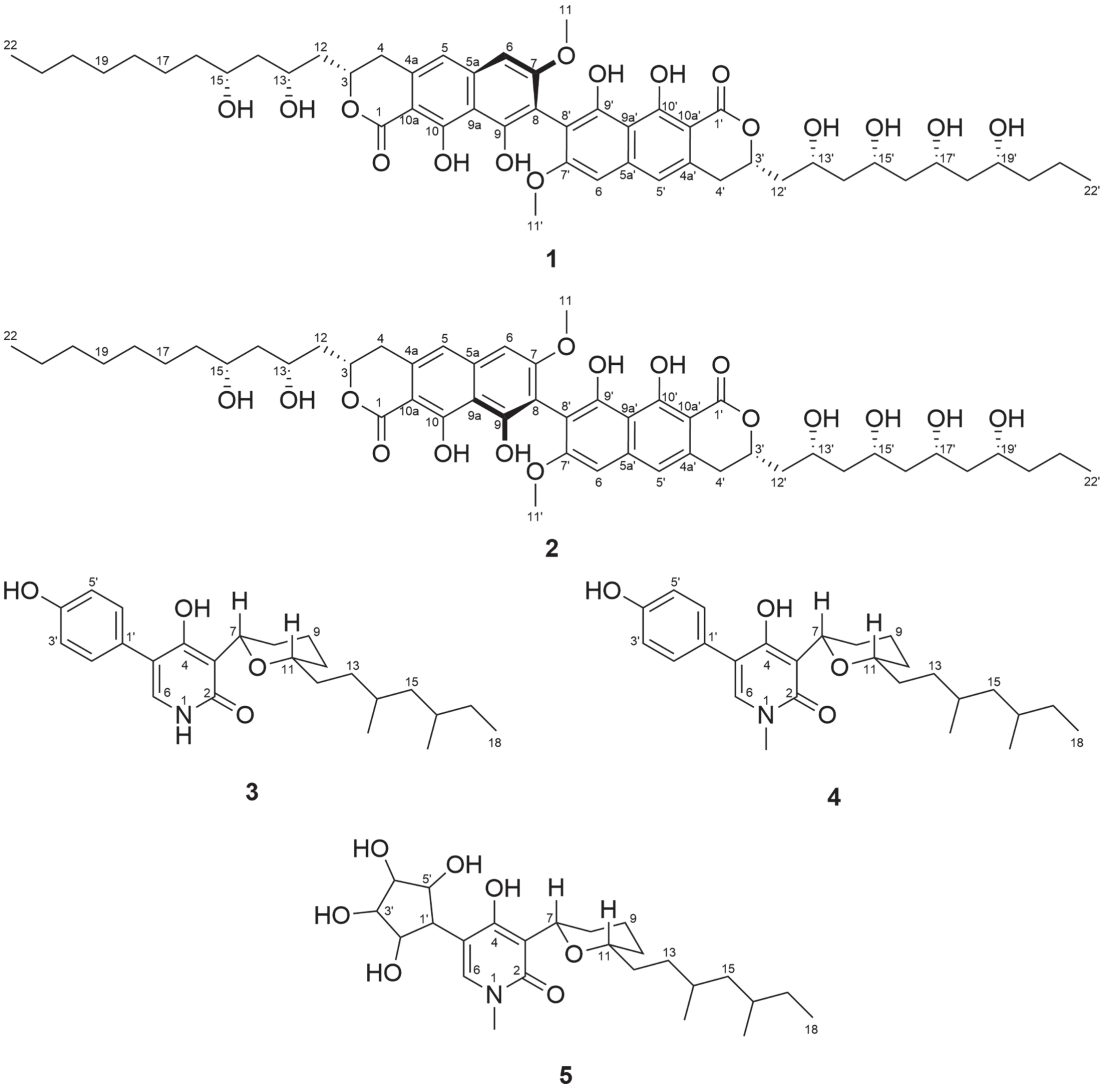
Chemical structures of isolated compounds 1‒5 in the co-culture system.

When designing a co-culture experiment, physical interactions or the small molecules produced by the microorganism are speculated to increase the amount of secondary metabolites produced in the co-culture system. To investigate the induction factors that enhanced the production of compounds in the co-culture, each of the three fungal strains was cultured with a disk containing acetone extracts of *Streptomyces* sp. 13F051 (0.5 mg) and analyzed by LC-MS. Except for one fungal strain (data not shown), compounds **1** and **2** that were induced in the co-culture of *M. schulzeri* 15F098 with *Streptomyces* sp. 13F051 and compounds **3-5** that were induced in the co-culture of *S. sphagnicola* 15S058 with *Streptomyces* sp. 13F051, were not detected in the culture system with acetone extracts of *Streptomyces* sp. 13F051, suggesting that these metabolites were induced by physical contacts with *Streptomyces* sp. 13F051 (Figs. 1, S1, and S3). Each of the two fungal strains was cultured with *Streptomyces* sp. KCB13F003 that does not produce trichostatins. The chemical profiles of each co-cultured extract were compared using LC-MS; compounds **1-5** were not observed in the co-culture system with *Streptomyces* sp. KCB13F003 (Figs. S2 and S4). However, because compounds **1-5** were not detected in the strain that does not produce trichostatins, it was concluded that these compounds were formed only by co-culture with *Streptomyces* sp. 13F051. Therefore, four compounds (**1-5**) induced by physical interaction with *Streptomyces* sp. 13F051 were further studied.

### Structure Determination

**Fig. 2:**
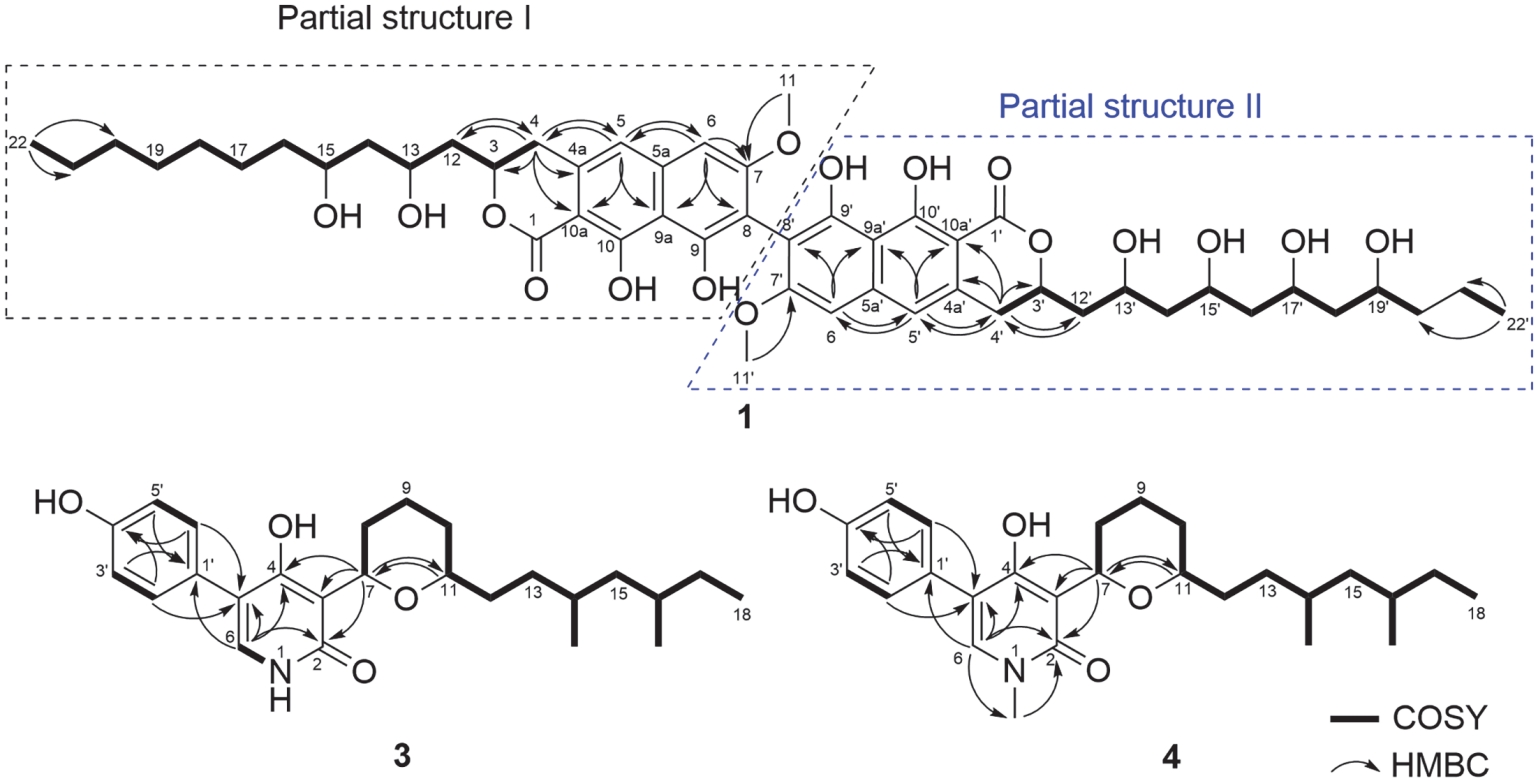
Selected COSY and HMBC correlations.

Mixture of compounds **1** and **2** was isolated as a brown amorphous powder. Based on NMR spectroscopy analysis, the planar structure of mixture was assumed to be dinapinone analogues isolated from *Talaromyces pinophilus* FKI-3864 [18-21]. Therefore, it was proposed that compounds produced by fungal strain *M. schulzeri* 15F098. A previous studies have reported that dinapinone analogues are atropisomers, and are separated by ODS C_30_ column [18-21]. Compounds **1** and **2** were obtained by further purification using ODS C_30_ column and confirmed using HRESIMS analysis and NMR spectroscopy analysis (Table 1). Molecular formulas of **1** and **2** were determined to be C_50_H_66_O_16_ with 18 unsaturated degrees. Similar to dinapinone derivatives, **1** was composed of two partial structures corresponding to 9,10-dihydroxy-7-methoxy-3-hydro-benzoisochromene-1-one and a linear hydroxylated alkyl chain. Based on the HMBC correlations between H/C-4 (*δ*_H/C_ 3.17 and 3.08/33.5) and H/C-12 (*δ*_H/C_ 2.19 and 2.02/42.5), the connection between dihydronaphthopyranone and the dihydroxylalkyl chain of partial structure I was determined as shown in Fig. 2 Additional HMBC correlations between H/C-4' (*δ*_H/C_ 3.17 and 3.08/33.5) and H/C-12' (*δ*_H/C_ 2.19 and 2.02/42.5) indicated that partial structure II consisted of dihydronaphthopyranone and tetrahydroxylalkyl chain. Furthermore, the connection pattern of the two partial structures was determined by the carbon chemical shifts of quaternary C-8 and C-8' (*δ*_C_ 109.5) (Fig. 2). The most similar structure of **1** was identified as dinapinone AC in previous study [21], which was not detected in co-culture system with *M. schulzeri* 15F098. The difference between the planar structure of **1** and dinapione AC is the long branched carbon chains with a propionate unit on both sides (C-12-C-22 and C-12'-C-22') instead of the short carbon chains (C-12-C-20 and C-12'-C-20') (Fig. 4). Among the dinapinone analogues, compound **1** had the longest branched carbon chain.

**Fig. 3:**
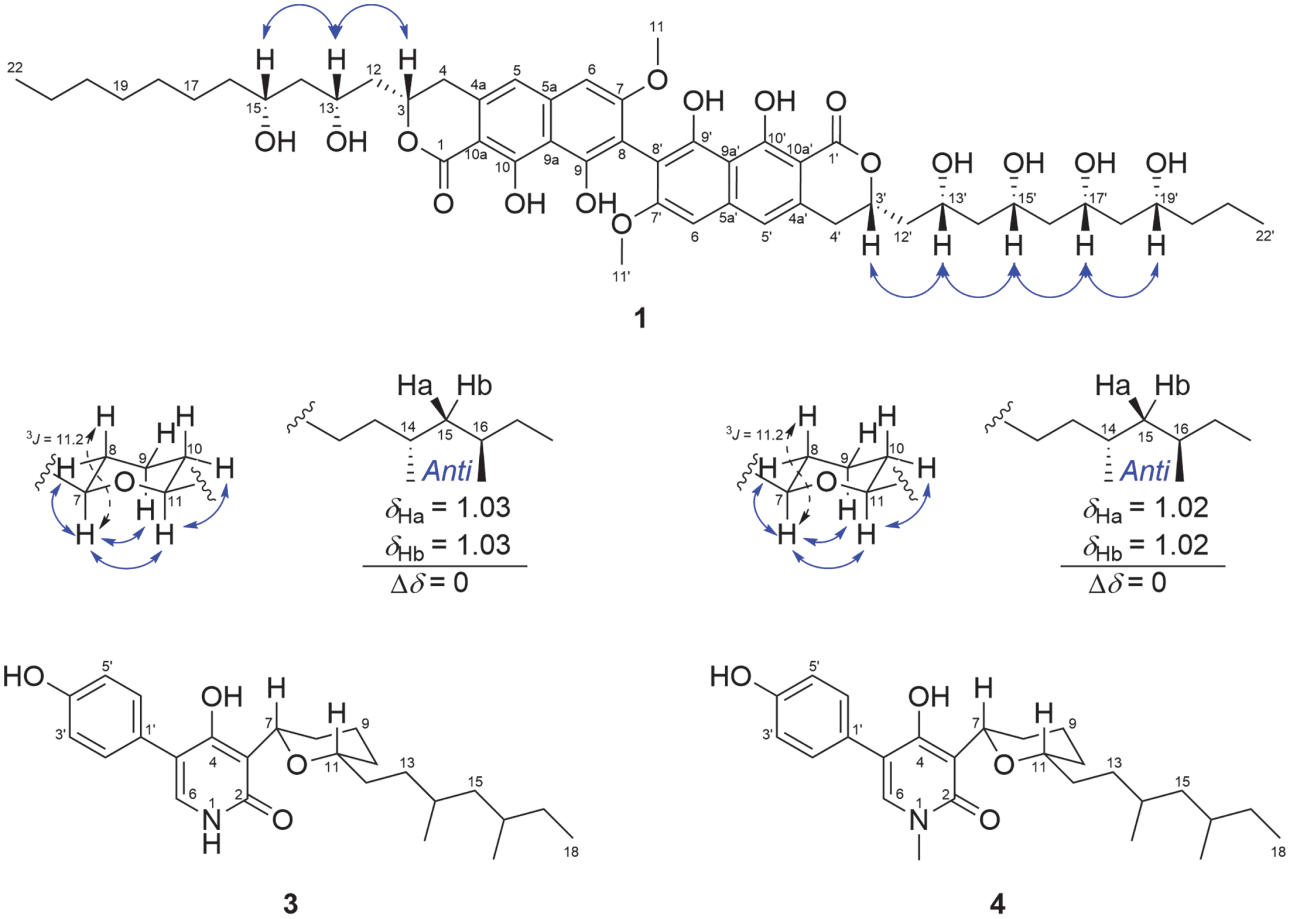
Key ROESY correlations and coupling constants.

The relative configurations of the hydroxylated C-3, C-13, and C-15 corresponding to carbon chains were established as 3S*, 13R*, and 15R* based on ROESY correlations (Fig. 3), and C-3', C-13', C-15', C-17', and C-19' had 3'S*, 13'R*, 15'R*, 17'R*, and 19'R* configuration, indicating that **1** had the same relative configuration as dinapione AC. Acetonide derivatizaion of the secondary alcohols in **1** did not yield sufficient amounts to confirm the relative configurations, presumably due to the presence of numerous secondary alcohols in **1**. According to previous reports, the CD data of dinapione analogues were compared with those of (*S*)-, (*R*)-vioxanthin, dihydronaphthopyranone to determine the absolute axis configuration. The *M*-axis configured substances exhibit negative specific rotation values, whereas *P*-axis compounds have positive specific rotation values [20, 21]. The specific rotation value of **1** was positive. Considering that **1** has structure similar to that of dinapione AC and has a positive value of specific rotation, the absolute axis configuration of **1** was determined as *P*.

While, NMR spectra of compound **2** were not obtained due to insufficient amount, compound **2** was determined as atropisomer of compound **1** based on HRESIMS spectroscopy. In addition, since **2** has negative specific rotation values, the absolute axis configuration of **2** was determined to be **M**.

**Fig. 4:**
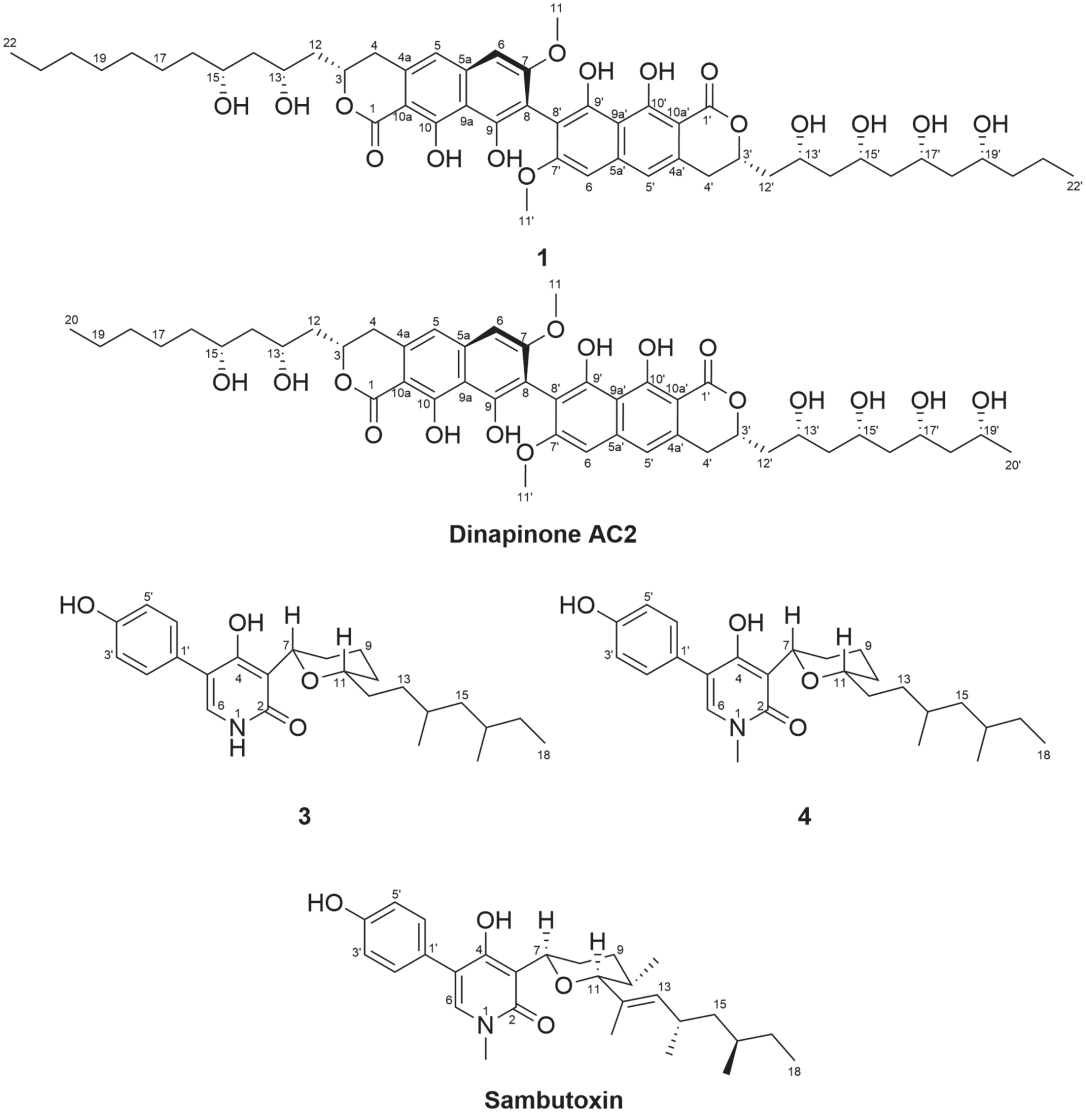
Structurally related known compounds, Dianapinone AC2 and Sambutoxin.

Compound **3** was isolated as a white amorphous powder. The molecular formula of **2** was determined to be C_25_H_35_NO_4_ with nine unsaturation degrees based on HRESIMS and NMR spectroscopy (Table 2). The NMR spectra of **3** suggested that **3** is a sambutoxin analogue, which was first isolated from the potato parasite *Fusarium sambucinum*[22]. Sambutoxin was also detected in co-culture system with *S. sphagnicola* 15S058, but NMR spectra were not obtained owing to insufficient amounts. Comparison of the ^1^H and ^13^C NMR spectra of **3** and sambutoxin indicated that olefin signals corresponding to the chain of sambutoxin were not detected in the spectra of **3**. Instead, two new signals were identified at *δ*_H/C_ 1.49/33.1 and *δ*_H/C_ 1.34 and 1.10/32.8, which were attributed to methylene. Furthermore, the three methyl signals substituted at N-1, C-10, and C-12 of sambutoxin were disappeared. Instead, NH signal was detected at *δ*_H_ 11.2 and two new methylene signals were detected at *δ*_H/C_ 1.65 and 1.30/30.6 and *δ*_H/C_ 1.49/33.1 corresponding to tetrahydronpyran and chain of **3**. Compound **3** is a sambutoxin analogue with demethylated tetrahydropyran and saturated carbon chain (Fig. 4). The planar structure of **3** was confirmed by ^1^H-^1^H correlation spectroscopy (COSY) and heteronuclear multiple bond correlation (HMBC) spectroscopy (Fig. 2).

The relative configuration of the tetrahydropyran moiety was determined using coupling constant and ROESY correlations (Fig. 3). The coupling constant between H-7 and H-8_ax_ was relatively large (^3^*J* = 11.2), indicating that the protons were located in axial position. Furthermore, the position of H-11 was established as axial by the rotating-frame nuclear Overhauser effect spectroscopy (ROESY) correlation between H-11 and H-7_ax_ and H-10_eq_. The relative configuration of the 1,3-dimethyl-barnched carbon chain was determined based on the ^1^H NMR chemical shift difference (Δδ) of its germinal methylene protons, H-15 (Fig. 3). The *anti*-configured substances have Δδ values of 0.0-0.2 ppm, while *syn*-compounds have Δδ values of 0.2-0.5 ppm [24]. The Δδ value between the germinal methylene protons (H-15) of **3** was relatively small, indicating that the two methyl groups of the 1,3-dimethyl-barnched carbon chain of **3** were in an anti-configuration.

Compound **4** was isolated as a white amorphous powder. The spectroscopic features of **4** were similar to those of **3**. However, its molecular formula was determined to be C_26_H_37_NO_4_ by HRESIMS, indicating the presence of a methyl group. The chemical composition of **4** was further confirmed by ^1^H and ^13^C NMR spectra, where an *N*-methyl signal was detected at *δ*_H/C_ 3.38/36.1, suggesting that the methyl group was substituted at the pyridone moiety. Based on COSY and HMBC correlations, the planar structure of **4** was identified as a demethyl sambutoxin analogue with a saturated carbon chain (Fig. 2) [23].

The relative configurations at C-7, C-11, C-14, and C-16 were determined based on the Δδ values of its germinal methylene protons (H-15) and ROESY correlation, similar to **3** as shown in Fig. 3 [23].

### Biological Activities

**Fig. 5:**
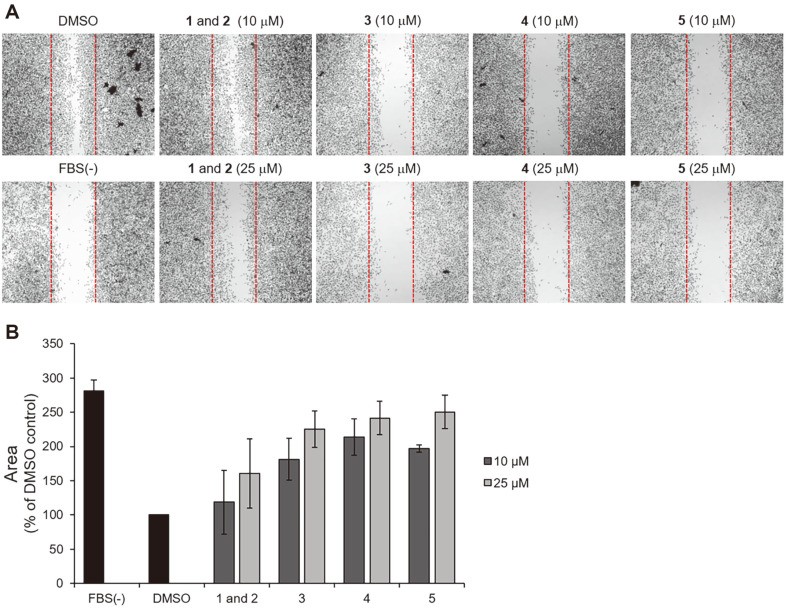
Effects of compounds on the migration of MDA-MB-231 cells. (**A**) Representative images of wound-healing assay. (**B**) Quantitative wound healing data obtained by measuring the wound area.

**Fig. 6:**
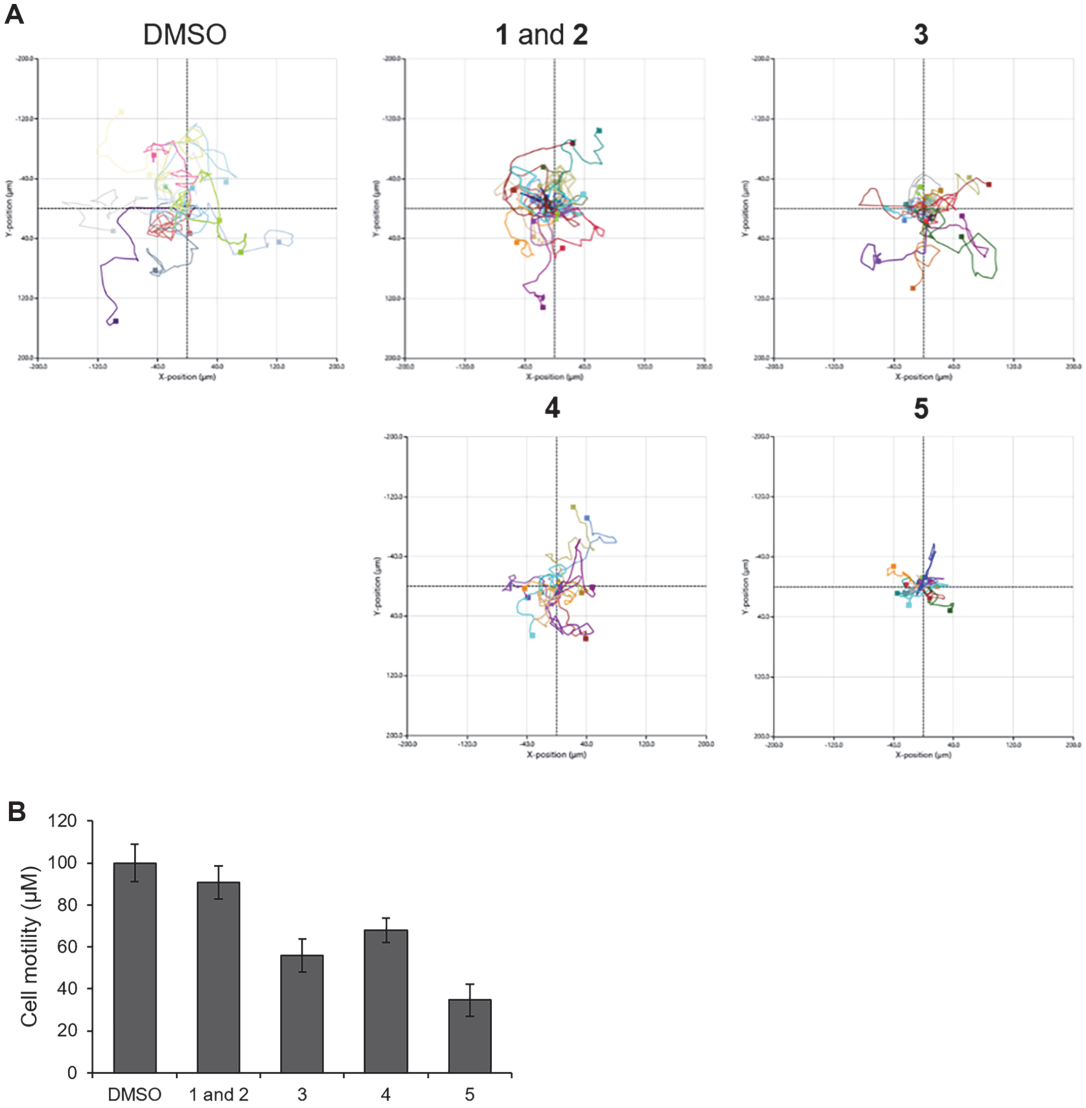
Effects of compounds on cell mobility of MDA-MB-231 cells. (**A**) Cells were treated with 25 μM of each compound. Cell movement was captured every 15 min for 12 h. (**B**) Cell mobility analyzed with HoloStudio M4 software.

Few microbial chemical cues promote the activation of silent biosynthetic gene clusters encoding defensive secondary metabolites to enhance survival in co-cultures of bacteria and fungi [10]. Based on the observation that the quantities of compounds **1** and **2** (mixture = 9:1, before separation of atropisomers) and **3-5** were enhanced by co-culture with actinomycete, it was assumed that **1** and **2** (mixture) and **3-5** have antibacterial activity. To test this hypothesis, an agar disk diffusion assay was performed to evaluate the antibacterial activity of **1** and **2** (mixture) and **3-5** against various pathogenic bacteria. However, compounds **1** and **2** (mixture) and **3-5** were inactive (Table S1). Compounds **1** and **2** (mixture) and **3-5** were not cytotoxic in various cancer cell lines (Table S2). Only 5 was cytotoxic against PC12 cells at a concentration of up to 50 μM. Cell migration is implicated in cancer cell metastasis [25]. To evaluate anti-migration activity, we performed a scratch wound healing assay in MDA-MB-231 cells. After treatment with the indicated concentrations of the test compounds for 24 h, the rate of cells migrating to the empty area was significantly decreased by treatment with compounds **3, 4**, and **5** in a dose-dependent manner (Fig. 5). To further investigate the effect of the compounds on cell migration, we performed time-lapse cell tracking analysis. The cells were treated with 25 μM of the indicated compounds. Cell movement was captured with a HoloMonitor M4 time-lapse cytometer every 15 min for 12 h. As expected, cell motility was markedly inhibited in cells treated with compounds **3** (44% inhibition), **4** (32% inhibition), and **5** (65% inhibition) compared to control cells treated with DMSO (Fig. 6).

## Discussion

This co-culture system, four new and one known compounds were isolated from the co-culture of two fungal strains with *Streptomyces* sp. 13F051. Based on comprehensive LC-MS analyses of chemical profiles and literature data, the four new compounds (**1-4**) originated from two fungal strains (*M. schulzeri* 15F098, and *S. sphagnicola* 15S058).

When the co-culture experiment was designed, the fungal secondary metabolites were expected to be produced by HDAC inhibitors produced by *Streptomyces* sp. 13F051 or by the interaction of physical contacts or chemicals other than trichostatins. Based on the disk diffusion assay with acetone extracts of *Streptomyces* sp. 13F051, it was found that compounds **1-5** were produced by physical interaction with *Streptomyces* sp. 13F051. The co-culture system led to complex microbial communities based on chemical-ecological interactions. Therefore, defining induction mechanisms is a challenging task. Only a few studies have described the induction mechanisms of secondary metabolites in co-culture system. Some studies have proposed that secondary metabolites are induced by the microbial defense system against the counterpart microorganism [10]. Only few examples have used microorganisms that produced enhancers of secondary metabolites, such as antibiotics, volatile organic compounds, quorum sensing molecules and mycolic acid [26]. However, there has been no reference to the use of microorganisms that produce HDAC inhibitors in co-culture systems. Although fungal derived compounds **1-5** were not produced by trichostatins, co-culture with a HDAC inhibitor producer is proposed as one of the strategies for inducing fungal secondary metabolites. Further broad-range co-culture experiments with fungal strains are require to confirm the effectiveness of this designed method.

The new compounds (**1-4**) structurally related to dinapione and sambutoxin. Although their structural novelty is not notable, the discovery of these compounds increases the structural diversity of available analogues and extends their biological activities.

## Supplemental Materials

Supplementary data for this paper are available on-line only at http://jmb.or.kr.



## Figures and Tables

**Table 1:** ^1^H (700 MHz) and ^13^C (150 MHz) NMR data of 1 in CD_3_OD:CDCl_3_ = 1:1.

Position	**1**		Position	**1**	
	*δ*_C_, type	*δ*_H_, mult. (*J* in Hz)		*δ*_C_, type	*δ*_H_, mult. (*J* in Hz)
1	172.4, C*^b^*		1'	172.4, C*^b^*	
2			2'		
3	78.7, CH	4.86, m	3'	78.7, CH	4.86, m
4	33.4, CH_2_	3.19, m 3.06, m	4'	33.4, CH_2_	3.19, m 3.06, m
4a	133.8, C		4a'	133.8, C	
5	117.3, CH	7.05, s	5'	117.3, CH	7.05, s
5a	141.0, C		5a'	141.0, C	
6	99.0, CH	6.76, s	6'	99.0, CH	6.76, s
7	162.6, C		7'	162.6, C	
8	109.0, C		8'	109.0, C	
9	155.8, C		9'	155.8, C	
9a	109.0, C		9a'	109.0, C	
10	163.8, C		10'	163.8, C	
10a	100.2, C		10a'	100.2, C	
11	56.4, CH_3_	3.82, s	11'	56.4, CH_3_	3.82, s
12	42.7, CH_2_	2.10, m 1.92, m	12'	42.7, CH_2_	2.10, m 1.92, m
13	68.6, CH	4.10, m*^a^*	13'	67.8, CH	4.10, m*^a^*
14	44.8, CH_2_	1.61, m*^a^*	14'	43.7, CH_2_	1.66, m*^a^* 1.59, m*^a^*
15	72.1, CH	3.80, m	15'	70.8, CH	4.05, m
16	38.6, CH_2_	1.44, m*^a^*	16'	44.2, CH_2_	1.67, m*^a^* 1.59, m*^a^*
17	26.0, CH_2_	1.42, m*^a^* 1.30, m*^a^*	17'	71.3, CH	4.03, m
18	30.3, CH_2_	1.24, m*^a^*	18'	44.3, CH_2_	1.59, m*^a^* 1.52, m
19	30.3, CH_2_	1.24, m*^a^*	19'	71.6, CH	3.77, m
20	32.5, CH_2_	1.25, m	20'	40.7, CH_2_	1.42, m*^a^*
21	23.3, CH_2_	1.27, m	21'	19.2, CH_2_	1.41, m*^a^* 1.34, m*^a^*
22	14.4, CH_3_	0.86, dd (8.5, 5.3)	22'	14.4, CH_3_	0.90, dd (8.5, 5.3)

^*a*^Resonances overlapped

^*b*^Peaks only detected in HMBC

**Table 2:** ^1^H (700 MHz) and ^13^C (150 MHz) NMR data of 3 and 4 in DMSO-*d*_6_.

Position	**3**		**4**	
	*δ*_C_, type	*δ*_H_, mult. (*J* in Hz)	*δ*_C_, type	*δ*_H_, mult. (*J* in Hz)
1		11.2, br		
2	160.9, C		160.1, C	
3	109.5, C		108.9, C	
4	161.8, C		160.6, C*^b^*	
5	113.1, C		112.8, C	
6	132.3, CH	7.11, s	137.0, C	7.55, s
7	76.7, CH	4.76, dd (11.2, 1.5)	77.1, CH	4.80, dd, (11.1, 1.5)
8	29.8, CH_2_	1.82, m*^a^* 1.41, m*^a^*	29.7, CH_2_	1.82, m*^a^* 1.39, m
9	22.7, CH_2_	1.82, m*^a^* 1.59, m	22.7, CH_2_	1.81, m*^a^* 1.59, m
10	30.6, CH_2_	1.65, m 1.30, m*^a^*	30.6, CH_2_	1.65, m 1.30, m*^a^*
11	79.2, CH	3.50, m	79.2, CH	3.49, m
12	33.1, CH_2_	1.49, m	33.1, CH_2_	1.50, m
13	32.8, CH_2_	1.34, m 1.10, m	32.8, CH_2_	1.34, m*^a^* 1.10, m*^a^*
14	29.6, CH	1.43, m	29.6, CH	1.43, m
15	43.7, CH_2_	1.03, t (7.0)	43.6, CH_2_	1.03, t (7.0)
16	31.1, CH	1.35, m*^a^*	31.1, CH	1.35, m*^a^*
17	29.7, CH_2_	1.25, m*^a^* 1.09, m	29.7, CH_2_	1.25, m*^a^* 1.09, m*^a^*
18	11.2, CH_3_	0.81, t (7.4)	11.2, CH_3_	0.81, t (7.4)
1-*N*-methyl			36.1, CH_3_	3.38, s
14-methyl	19.4, CH_3_	0.80, d (6.5)	19.4, CH_3_	0.80, d (6.5)
16-methyl	18.8, CH_3_	0.78, d (6.5)	18.8, CH_3_	0.78, d (6.5)
1'	124.7, C		124.5, C	
2'	130.0, CH	7.19, d (8.4)	130.0, CH	7.21, d (8.5)
3'	114.8, CH	6.73, d (8.4)	114.8, CH	6.75, d (8.5)
4'	156.4, C		156.4, C	
5'	114.8, CH	6.73, d (8.4)	114.8, CH	6.75, d (8.5)
6'	130.0, CH	7.19, d (8.4)	130.0, CH	7.21, d (8.5)

^*a*^Resonances overlapped

^*b*^Peaks only detected in HMBC NMR
