# Supplementary material for: Induction of Fungal Secondary Metabolites by Co-Culture with Actinomycete Producing HDAC Inhibitor Trichostatins
Source: J Microbiol Biotechnol. 2023 Jul 26;33(11):1437–47. doi: 10.4014/jmb.2301.01017 (PMC10699267; doi:10.4014/jmb.2301.01017)
Supplement: Supplementary file 1 [file jmb-33-11-1437-supple.pdf]

SUPPORTING INFORMATION

Induction of Fungal Secondary Metabolites by Co-culture with Actinomycete Producing HDAC Inhibitor Trichostatins

Gwi Ja Hwang,[1] Jongtae Roh,[1,2] Sangkeun Son,[4] Byeongsan Lee,[1] Jun-Pil Jang,[1] Jae-Seoun Hur,[3] Young-Soo Hong,[1,2] Jong Seog Ahn,[1,2] Sung-Kyun Ko,[1,2]* and Jae-Hyuk Jang[1,2]*

[1]Chemical Biology Research Center, Korea Research Institute of Bioscience and Biotechnology (KRIBB), Cheongju 28116, South Korea

[2]Department of Biomolecular Science, KRIBB school of Bioscience, Korea University of Science and Technology (UST), Daejeon 34141, South Korea

[3]Korean Collection for Type Cultures (KCTC), Biological Resource Center, Korea Research Institute of Bioscience and Biotechnology (KRIBB), Jeongeup 56212, South Korea

[4]Present address: Antimicrobial Discovery Center, Department of Biology, Northeastern University, Boston 02115 MA, USA

Contents

Table S1. Antibacterial test of mixture (**1** and **2**) and **3**–**5**

Table S2. Cell Viability test of mixture (**1** and **2**) and **3**–**5**

Figure S1. LC-MS profiles of cultured extracts of *Streptomyces* sp. 13F051 and *Myrmecridium schulzeri* 15F098

Figure S2. LC-MS profiles of cultured extracts of *Streptomyces* sp. 13F003 and *Myrmecridium schulzeri* 15F098

Figure S3. LC-MS profiles of cultured extracts of *Streptomyces* sp. 13F051 and *Scleroconidioma sphagnicola* 15S058

Figure S4. LC-MS profiles of cultured extracts of *Streptomyces* sp. 13F003 and *Scleroconidioma sphagnicola* 15S058

Figure S5. ^{1}H NMR spectrum (700 MHz) of **1** in CD_3OD:$CDCl_3$ = 1:1

Figure S6. ^{13}C NMR spectrum (175 MHz) of **1** in CD_3OD:$CDCl_3$ = 1:1

Figure S7. COSY spectrum of **1** in CD_3OD:$CDCl_3$ = 1:1

Figure S8. HSQC-DEPT spectrum of **1** in CD_3OD:$CDCl_3$ = 1:1

Figure S9. HMBC spectrum of **1** in CD_3OD:$CDCl_3$ = 1:1

Figure S10. ROESY spectrum of **1** in CD_3OD:$CDCl_3$ = 1:1

Figure S11. HRESIMS spectrum of **1**

Figure S12. HRESIMS spectrum of **2**

Figure S13. ^{1}H NMR spectrum (700 MHz) of **3** in DMSO-d_6

Figure S14. ^{13}C NMR spectrum (175 MHz) of **3** in DMSO-d_6

Figure S15. COSY spectrum of **3** in DMSO-d_6

Figure S16. HSQC-DEPT spectrum of **3** in DMSO-d_6

Figure S17. HMBC spectrum of **3** in DMSO-d_6

Figure S18. ROESY spectrum of **3** in DMSO-d_6

Figure S19. HRESIMS spectrum of **3**

Figure S20. ^{1}H NMR spectrum (700 MHz) of **4** in DMSO-d_6

Figure S21. ^{13}C NMR spectrum (175 MHz) of **4** in DMSO-d_6

Figure S22. COSY spectrum of **4** in DMSO-d_6

Figure S23. HSQC-DEPT spectrum of **4** in DMSO-d_6

Figure S24. HMBC spectrum of **4** in DMSO-d_6

Figure S25. ROESY spectrum of **4** in DMSO-d_6

Figure S26. HRESIMS spectrum of **4**
Figure S27. ^{1}H NMR spectrum (700 MHz) of **5** in DMSO-d_6
Figure S28. LRESIMS spectrum of **5**

Table S1 Antibacterial test of mixture (**1** and **2**) and **3–5**

	Strain	Zone of inhibition (Diameter in mm)	
		Mixture (**1** and **2**) and **3–5**[a]	Ciprofloxacin[b]
Gram positive	*Enterococcus faecalis* KCTC 5191	-	25
	Bacillus subtilis KCTC 1021	-	38
Gram negative	*Escherichia coli* CCARM 1356	-	36
	Pseudomonas aeruginosa KCTC 2004	-	27

[a]50 μg/disk
[b]10 μg/disk, positive control

Table S2. Cell viability test of mixture (**1** and **2**) and **3–5**

Cell line	IC_{50} value (μM)				
	Mixture (**1** and **2**)	**3**	**4**	**5**	Doxorubicin[a]
MDA-MB-231	> 50	> 50	> 50	> 50	1.85
HeLa	> 50	> 50	> 50	> 50	1.318
Neuro2a	> 50	> 50	> 50	> 50	0.639
PC12	> 50	> 50	> 50	> 50	2.29

[a]Positive control

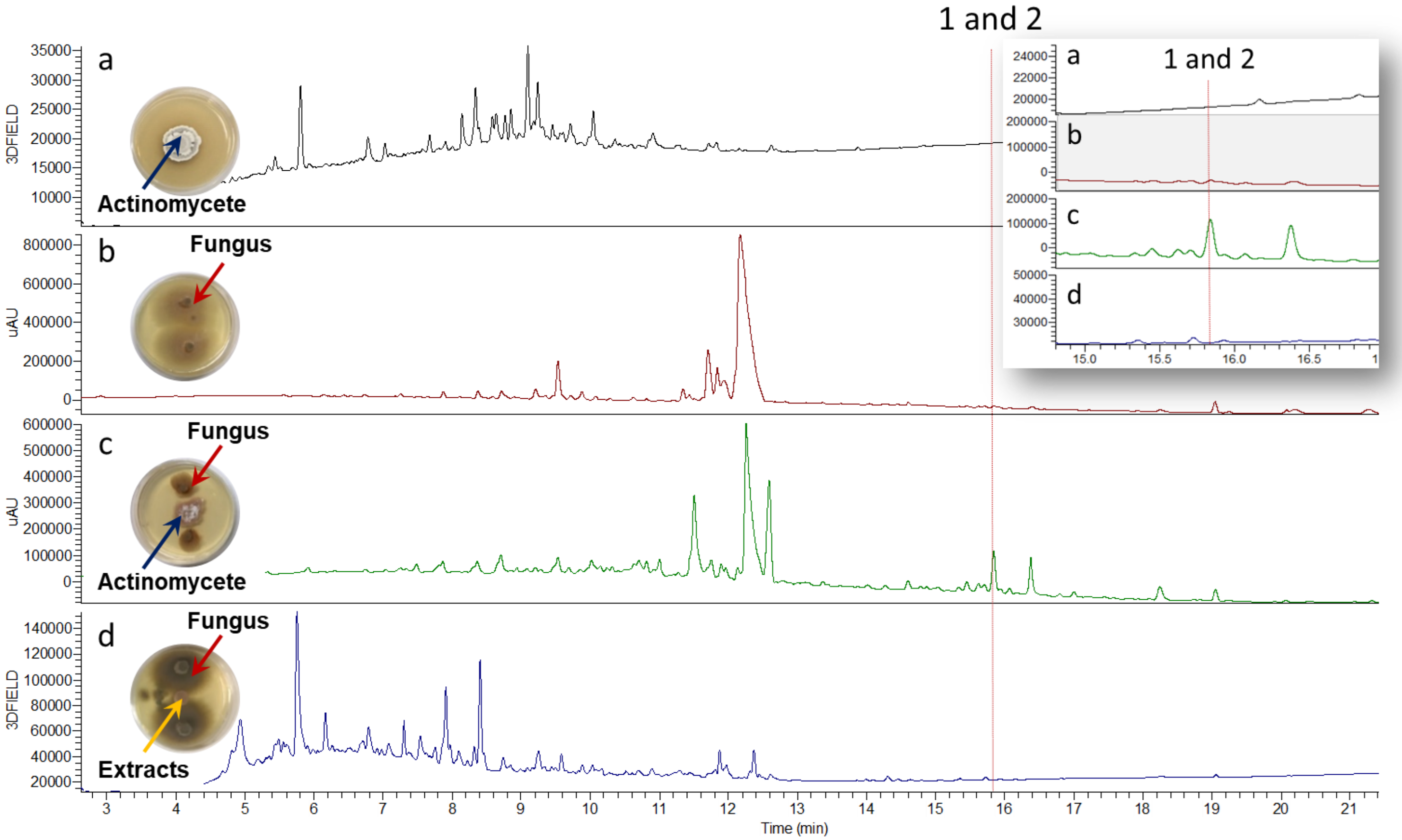

Figure S1. LC-MS profiles of mono-cultured extracts of *Streptomyces* sp. 13F051 (a), mono-cultured extracts of *Myrmecridium schulzeri* 15F098 (b), co-cultured extracts of *Streptomyces* sp. 13F051 and *Myrmecridium schulzeri* 15F098 (c), and mono-cultured extracts of *Myrmecridium schulzeri* 15F098 loaded with a paper disk containing the cultured extracts of *Streptomyces* sp. 13F051 (d). Blue arrow: actinomycete growing colony; red arrow: fungal strain growing colony; yellow arrow: the paper disk. Panel on the right is enlarged LC-MS data which part is induction peak of compounds **1** and **2**.

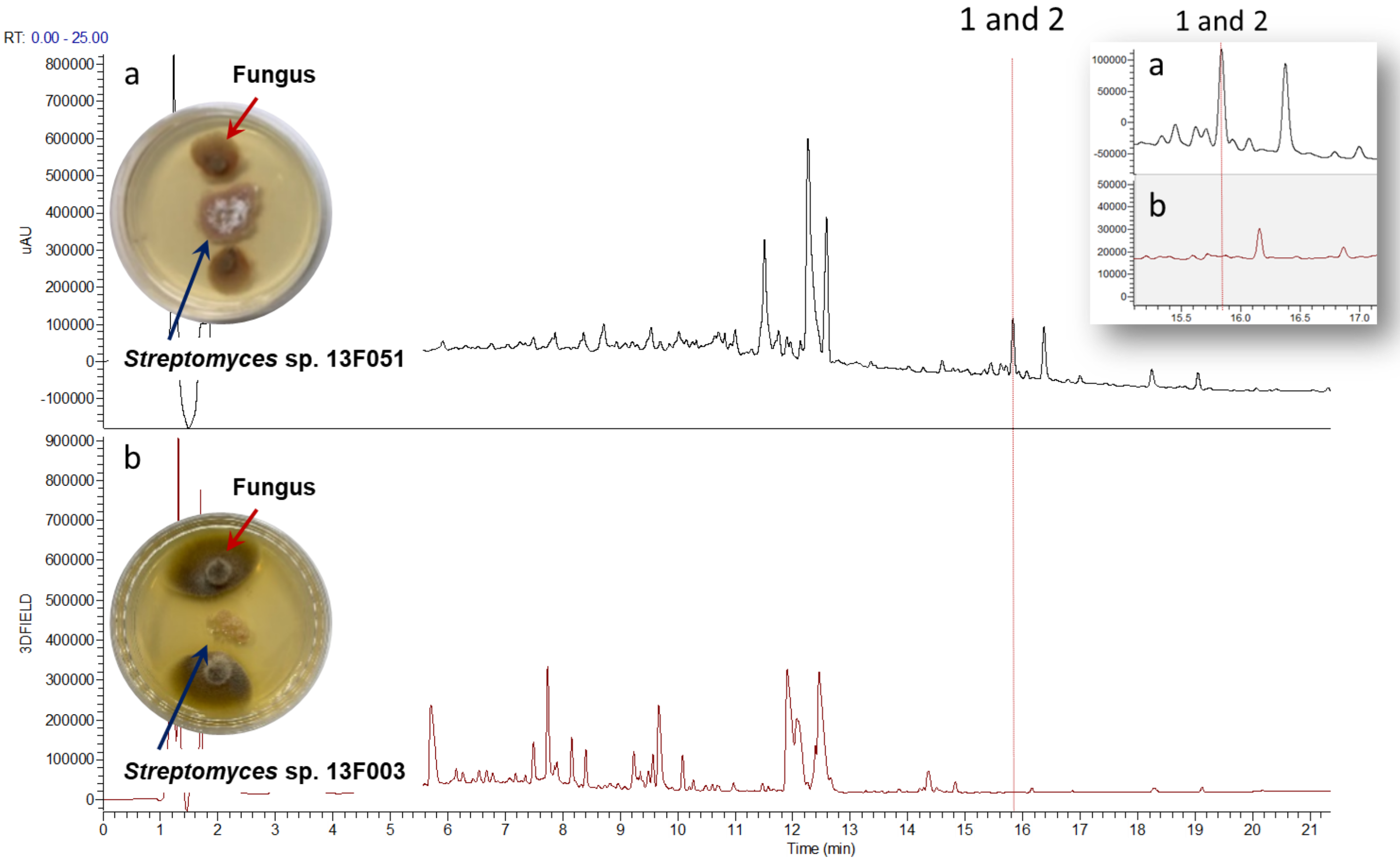

Figure S2. LC-MS profiles of co-cultured extracts of *Streptomyces* sp. 13F051 and *Myrmecridium schulzeri* 15F098 (a), co-cultured extracts of *Streptomyces* sp. 13F003 and *Myrmecridium schulzeri* 15F098 (b). Blue arrow: actinomycete growing colony; red arrow: fungal strain growing colony. Panel on the right is enlarged LC-MS data which part is induction peaks of compounds **1** and **2**.

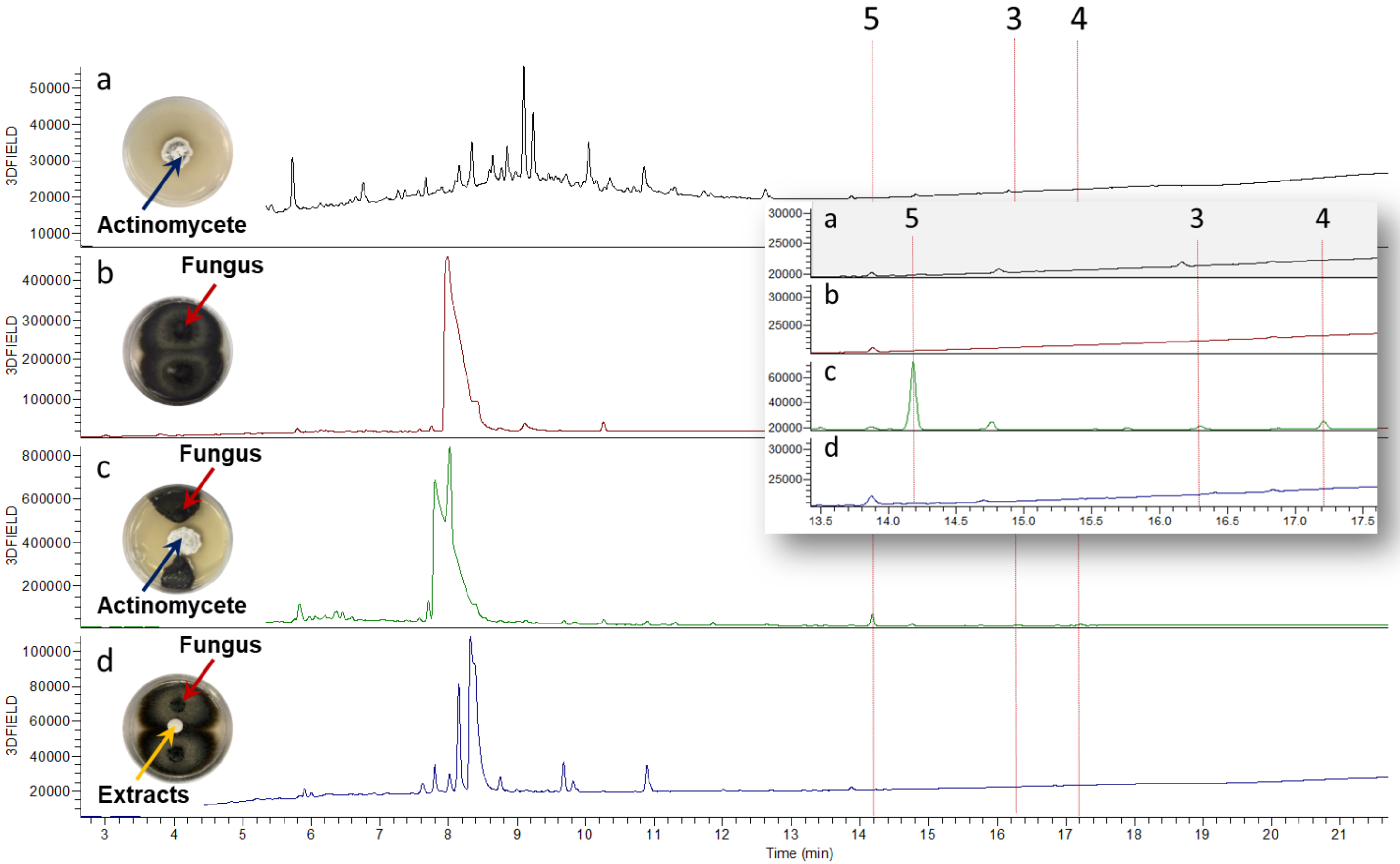

Figure S3. LC-MS profiles of mono-cultured extracts of *Streptomyces* sp. 13F051 (a), mono-cultured extracts of *Scleroconidioma sphagnicola* 15S058 (b), co-cultured extracts of *Streptomyces* sp. 13F051 and *Scleroconidioma sphagnicola* 15S058 (c), and mono-cultured extracts of *Scleroconidioma sphagnicola* 15S058 loaded with a paper disk containing the cultured extracts of *Streptomyces* sp. 13F051 (d). Blue arrow: actinomycete growing colony; red arrow: fungal strain growing colony; yellow arrow: the paper disk. Panel on the right is enlarged LC-MS data which part is induction peaks of compounds **3**–**5**.

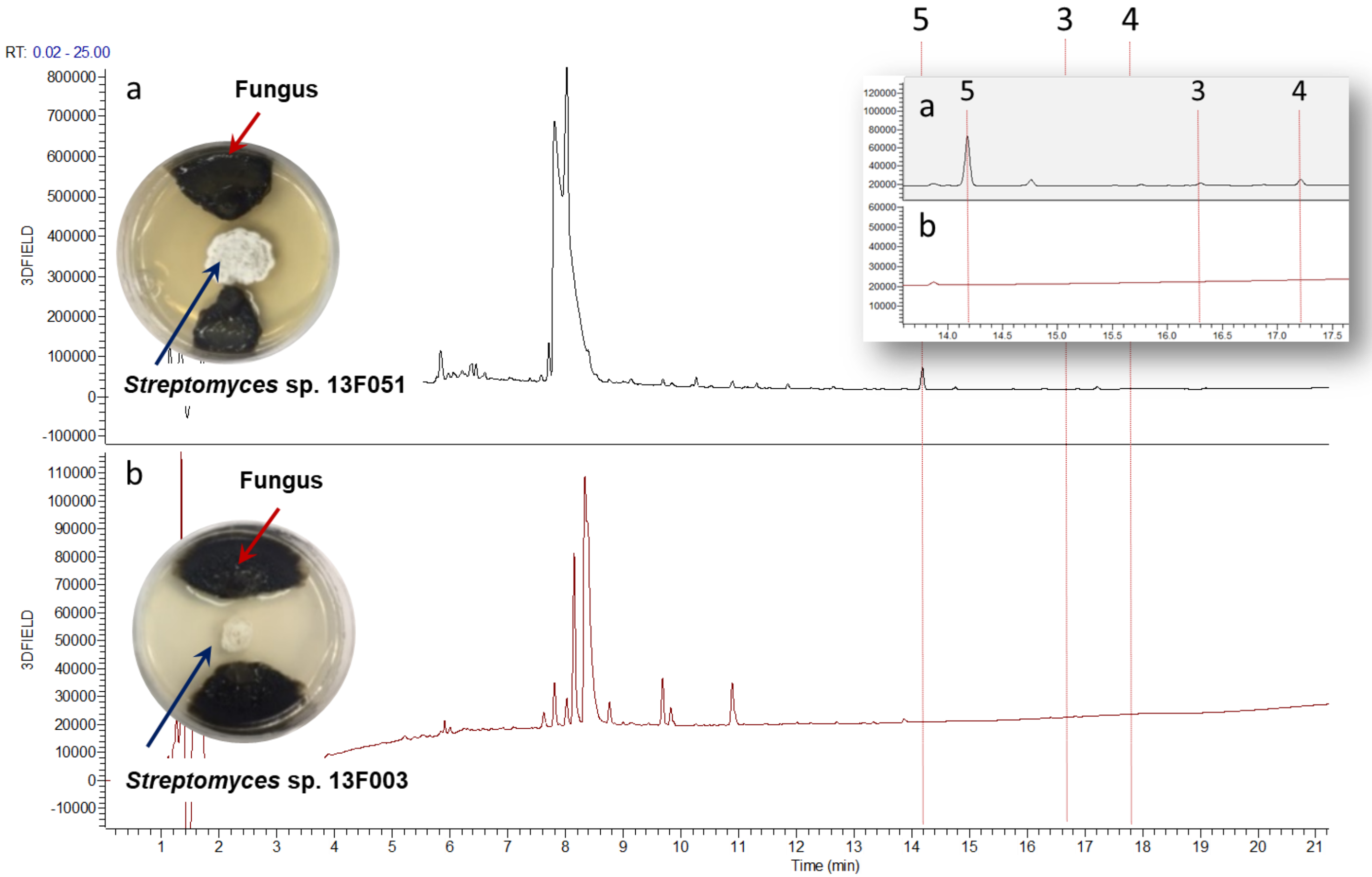

Figure S4. LC-MS profiles of co-cultured extracts of *Streptomyces* sp. 13F051 and *Scleroconidioma sphagnicola* 15S058 (a), co-cultured extracts of *Streptomyces* sp. 13F003 and *Scleroconidioma sphagnicola* 15S058 (b). Blue arrow: actinomycete growing colony; red arrow: fungal strain growing colony. Panel on the right is enlarged LC-MS data which part is induction peaks of compounds **3**–**5**.

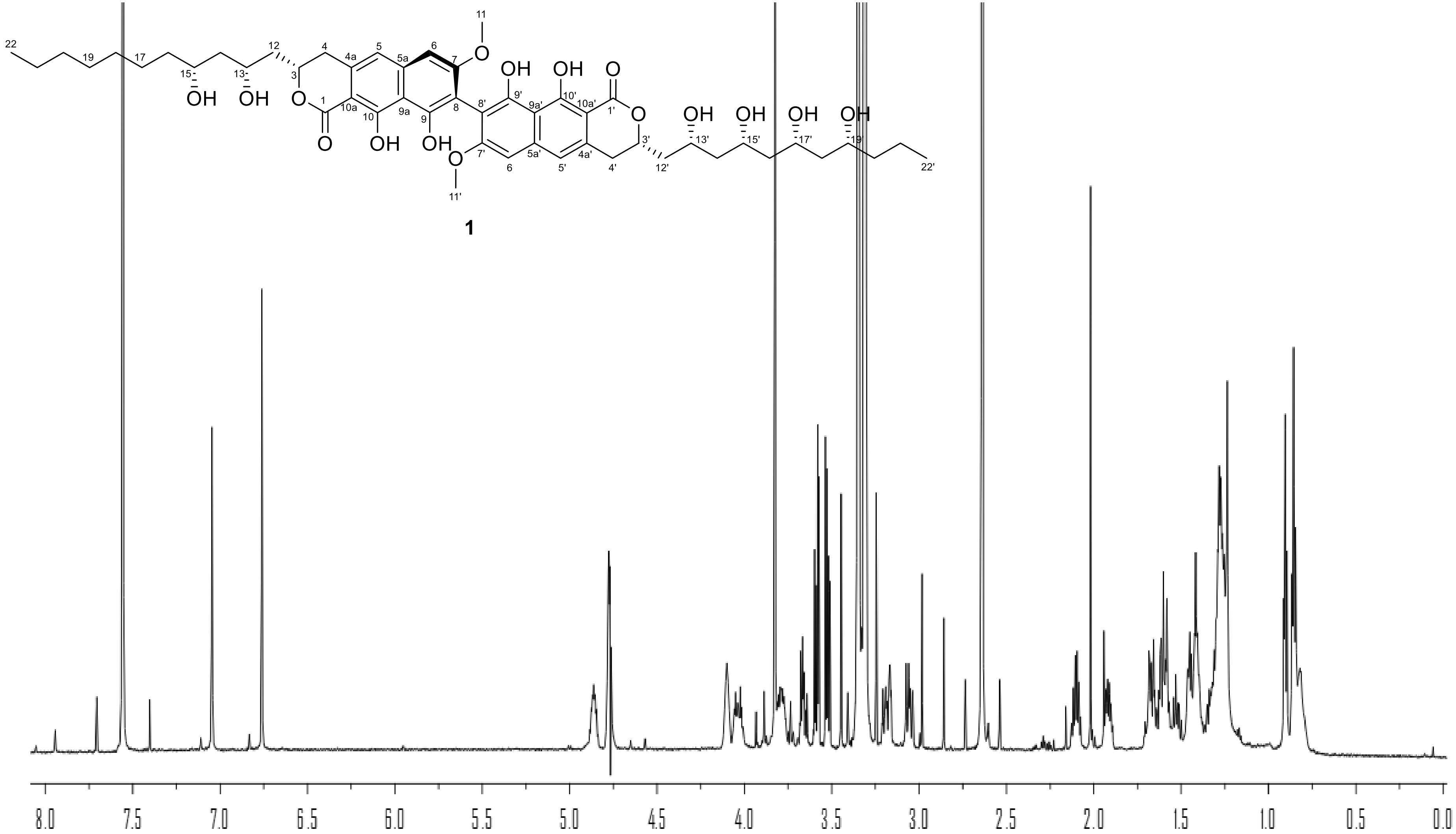

Figure S5. ^{1}H NMR spectrum (700 MHz) of **1** in $CD_3OD:CDCl_3$ = 1:1

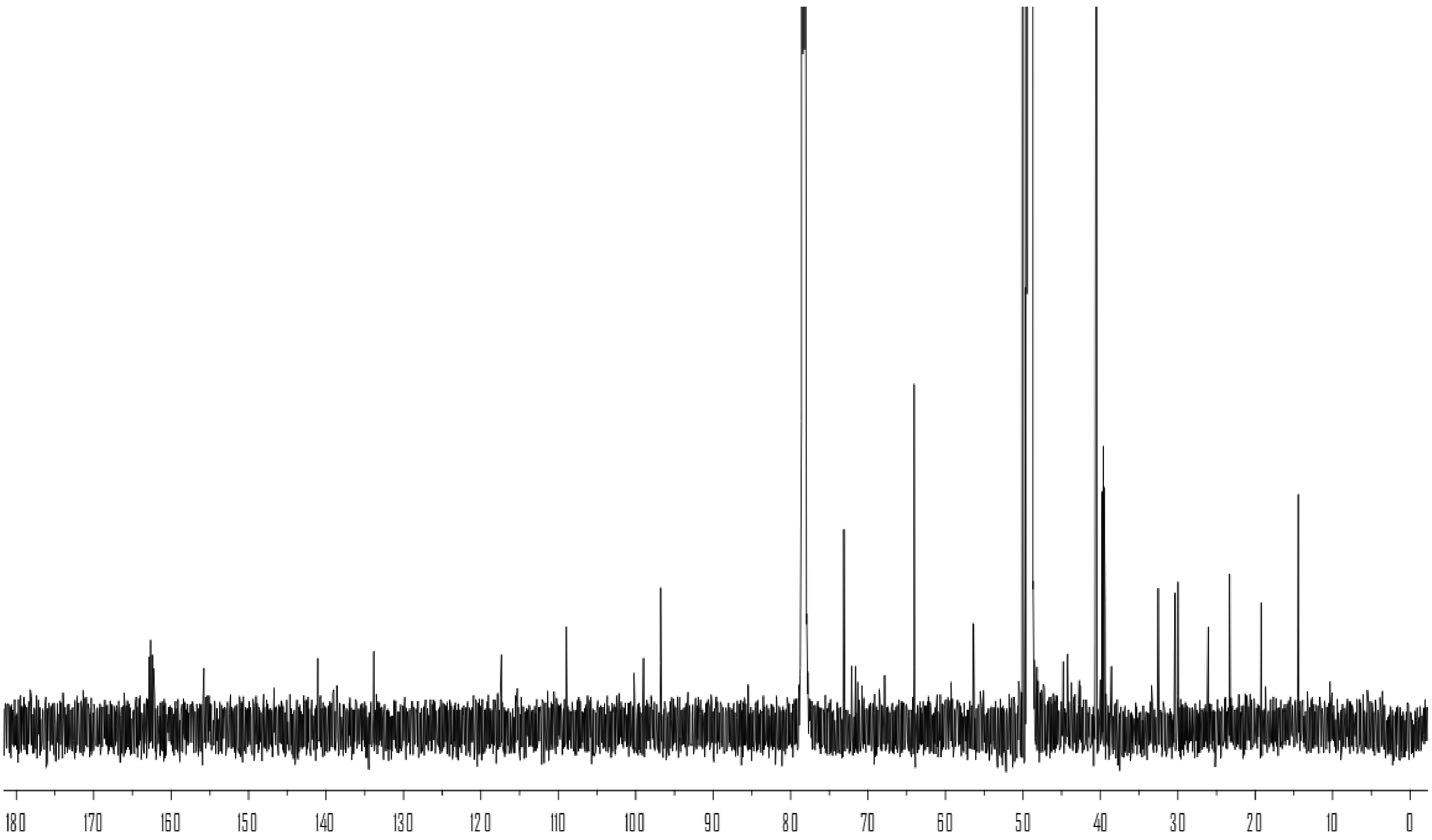

Figure S6. ^{13}C NMR spectrum (175 MHz) of **1** in $CD_3OD:CDCl_3$ = 1:1

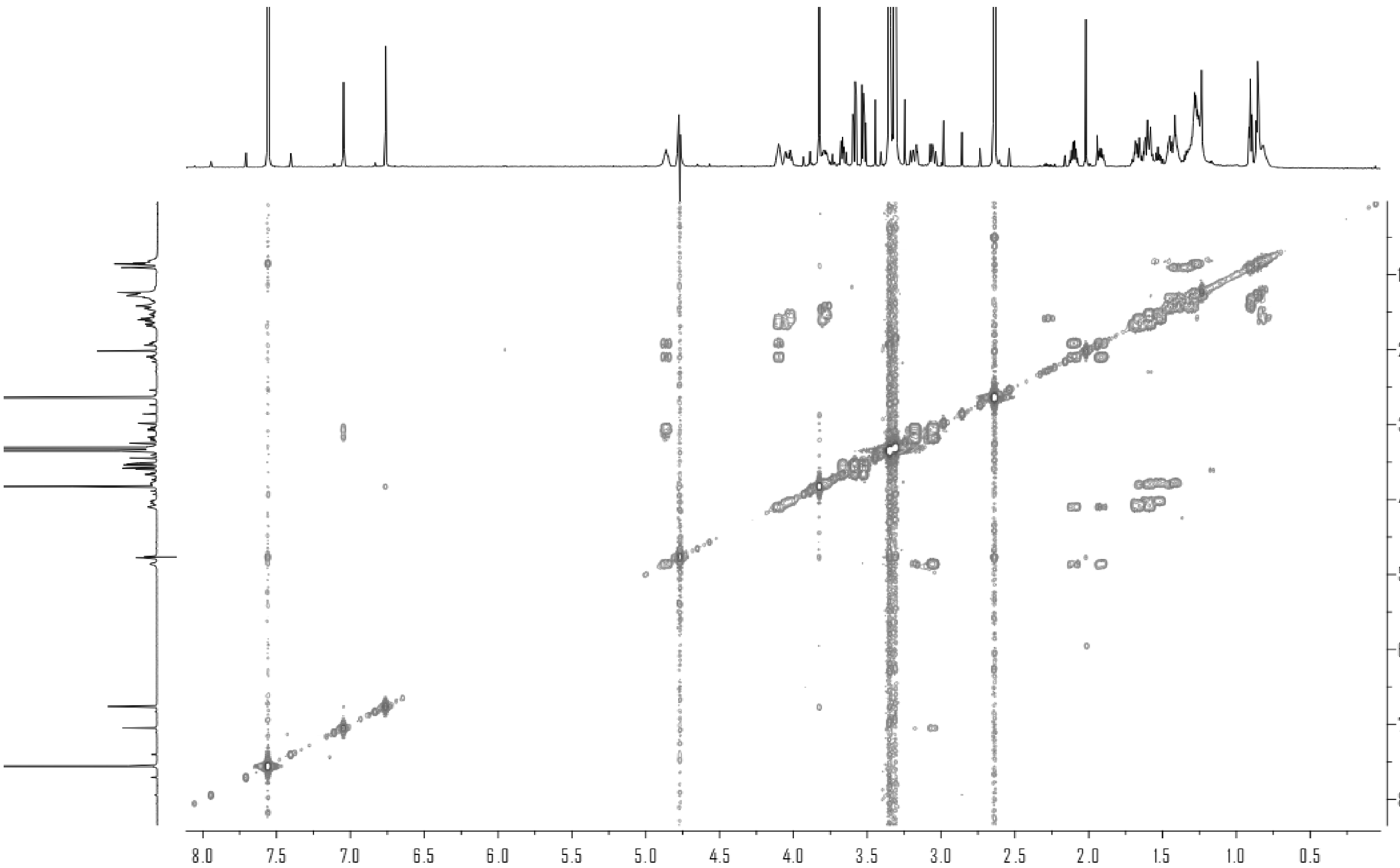

Figure S7. COSY spectrum of **1** in CD_3OD:$CDCl_3$ = 1:1

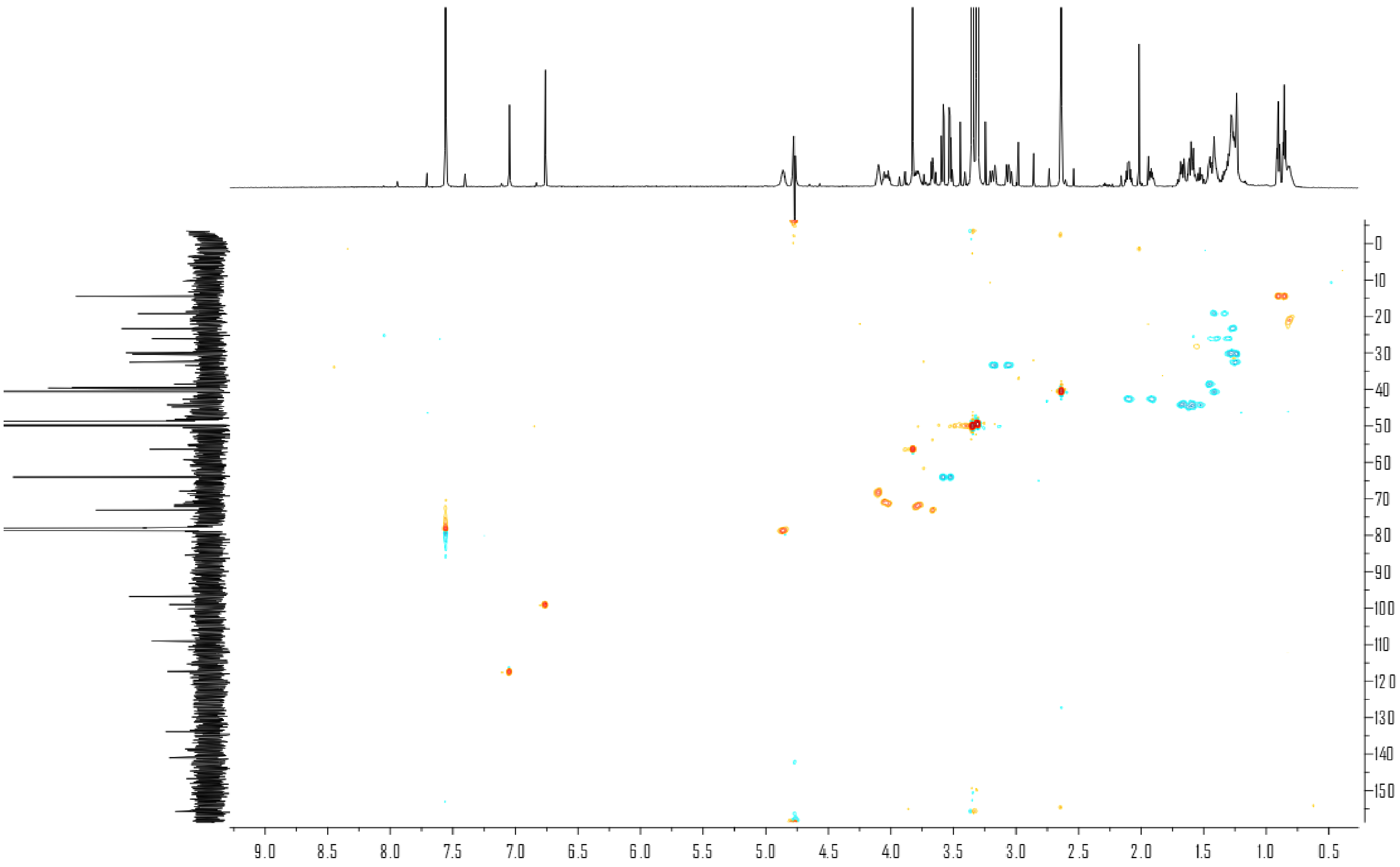

Figure S8. HSQC-DEPT spectrum of **1** in CD_3OD:$CDCl_3$ = 1:1

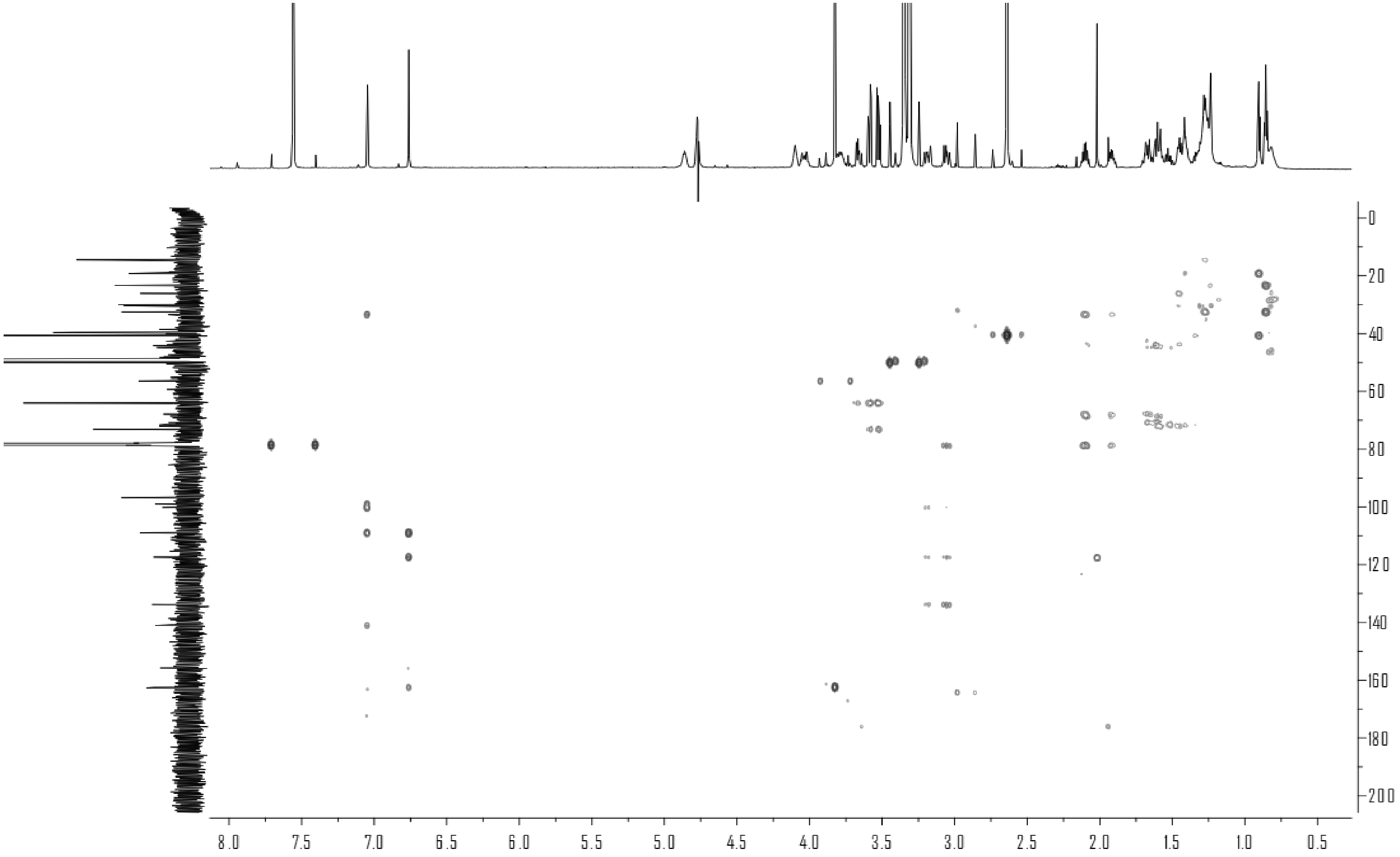

Figure S9. HMBC spectrum of **1** in $CD_3OD:CDCl_3$ = 1:1

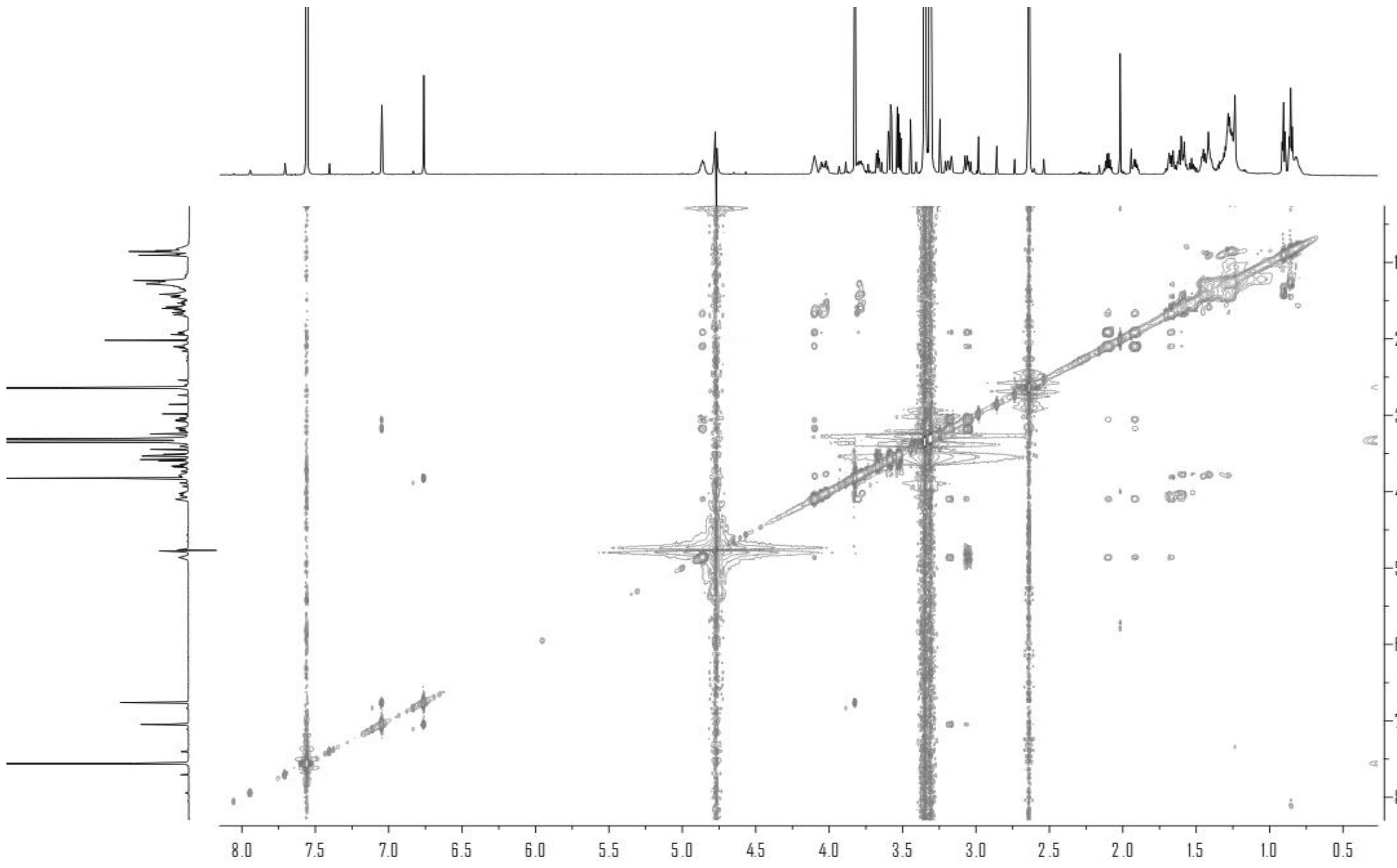

Figure S10. ROESY spectrum of **1** in $CD_3OD:CDCl_3$ = 1:1

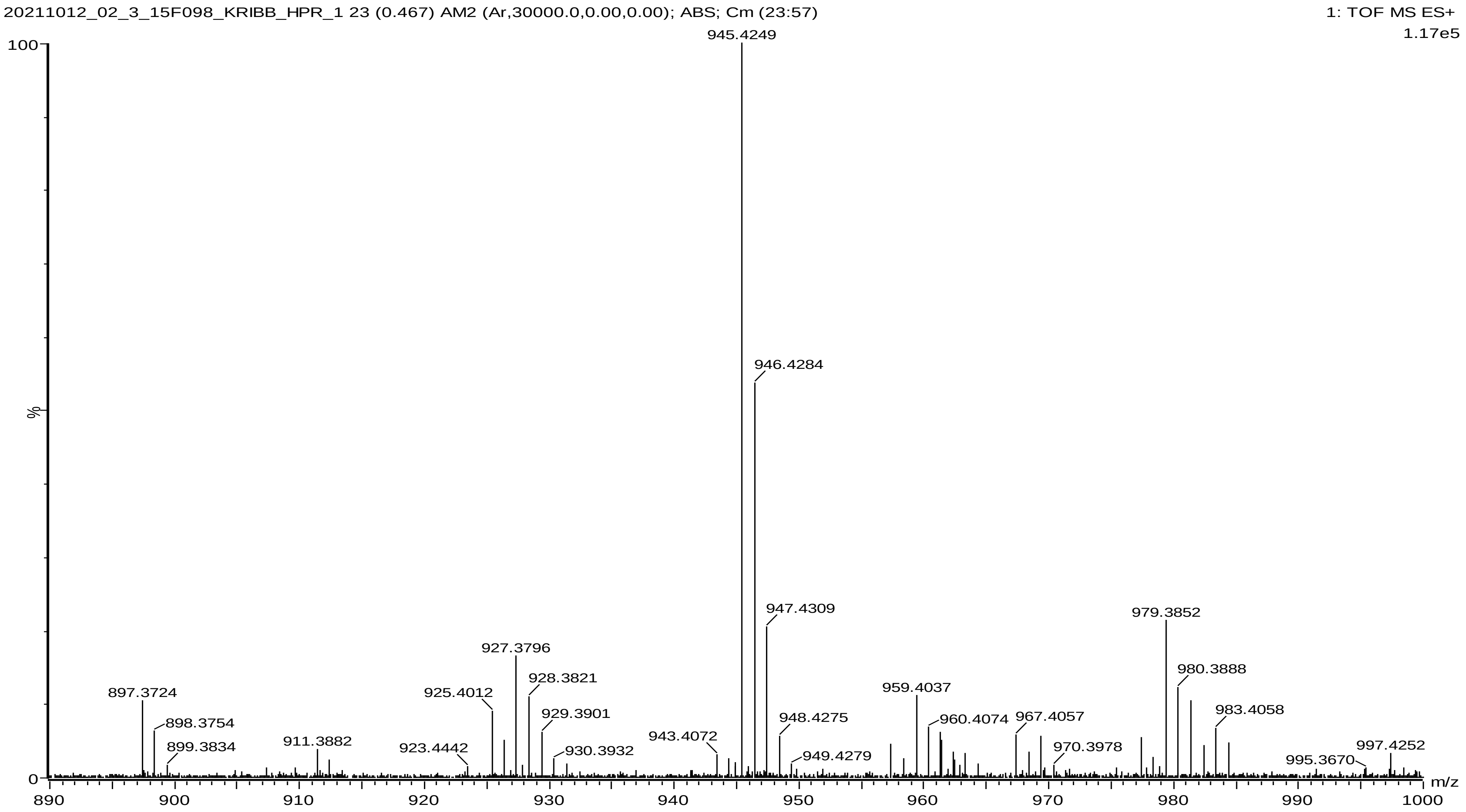

Figure S11. HRESIMS spectrum of **1**

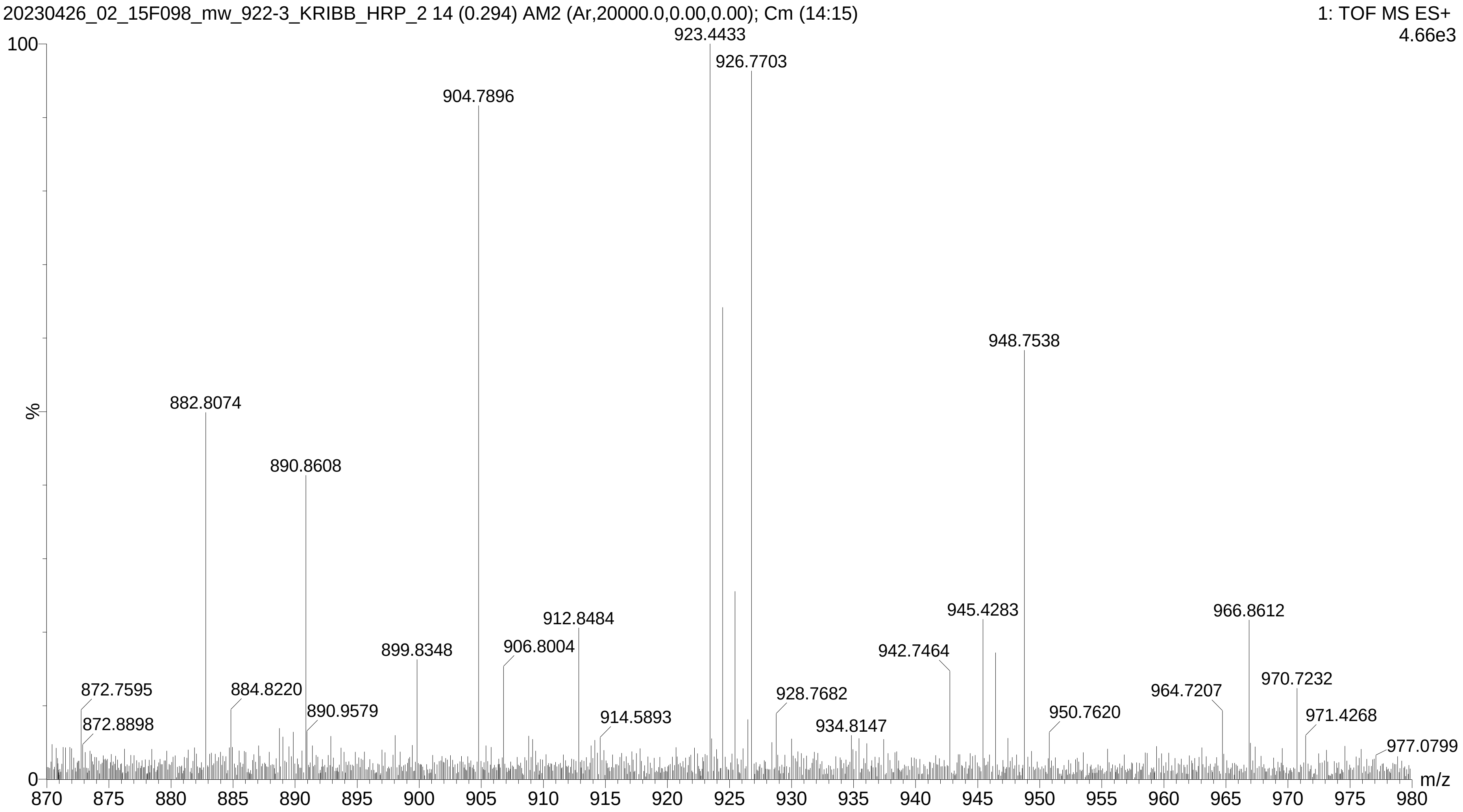

Figure S12. HRESIMS spectrum of **2**

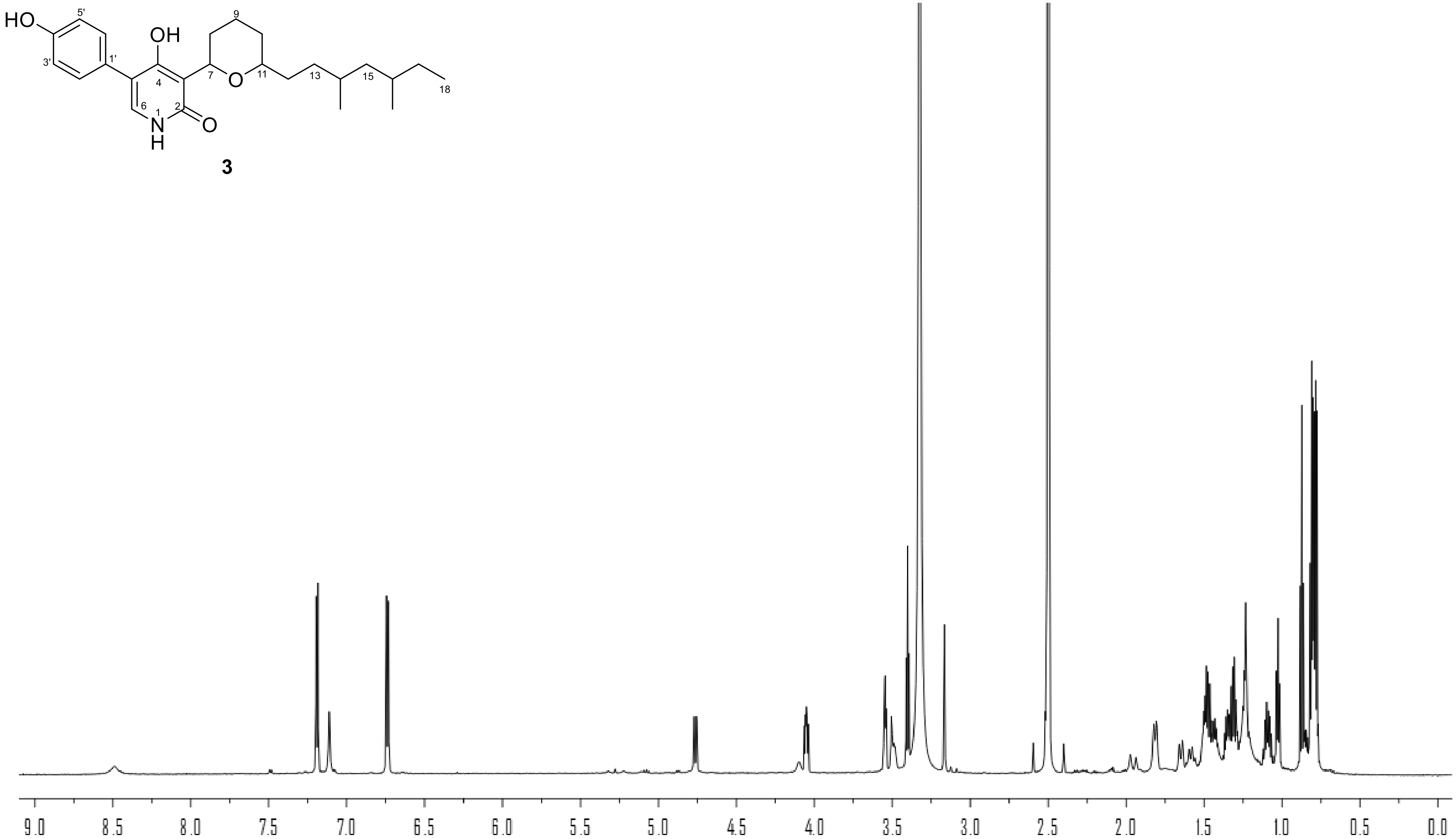

Figure S13. ^{1}H NMR spectrum (700 MHz) of **3** in DMSO-d_6

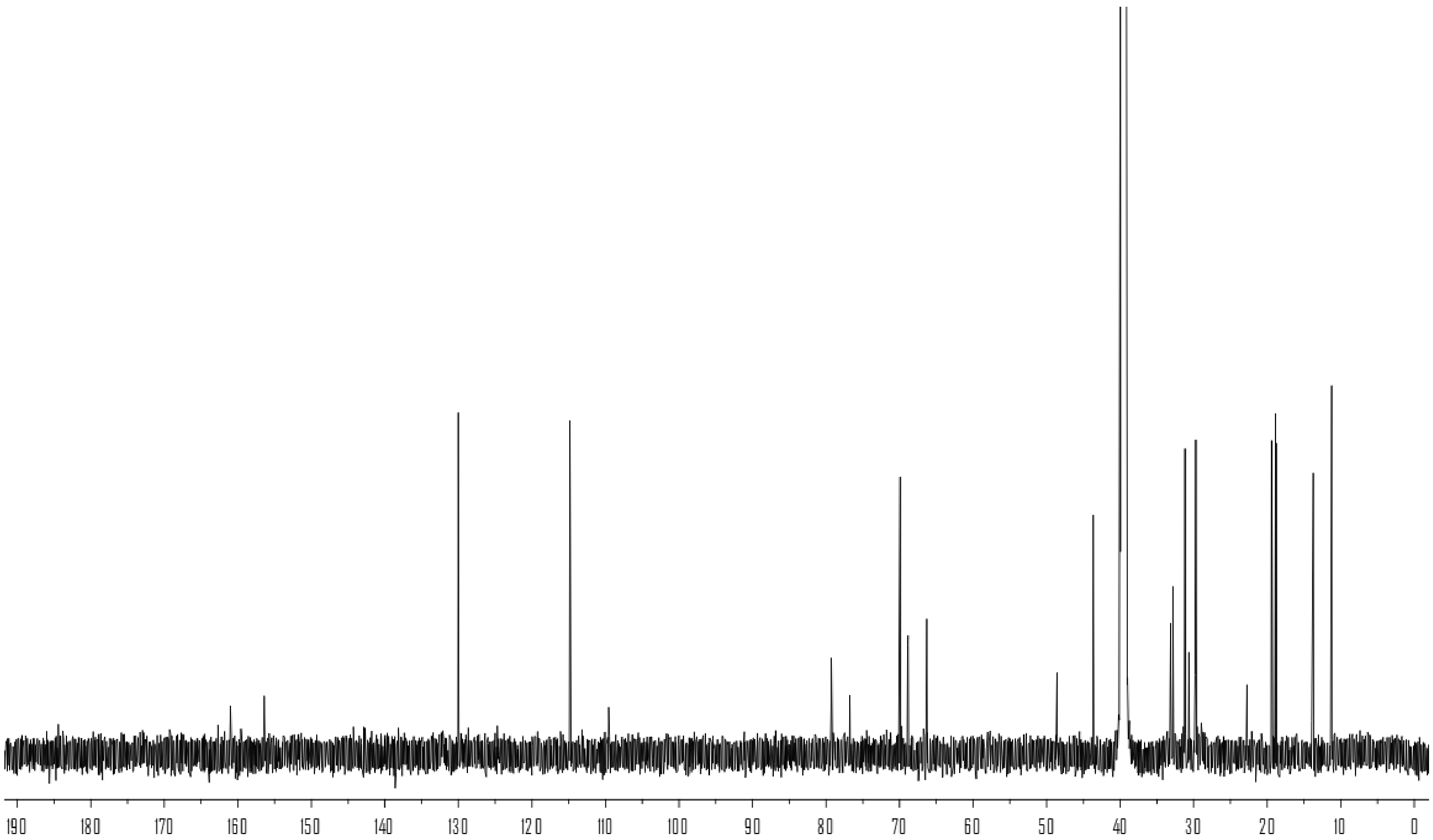

Figure S14. ^{13}C NMR spectrum (175 MHz) of **3** in DMSO-d_6

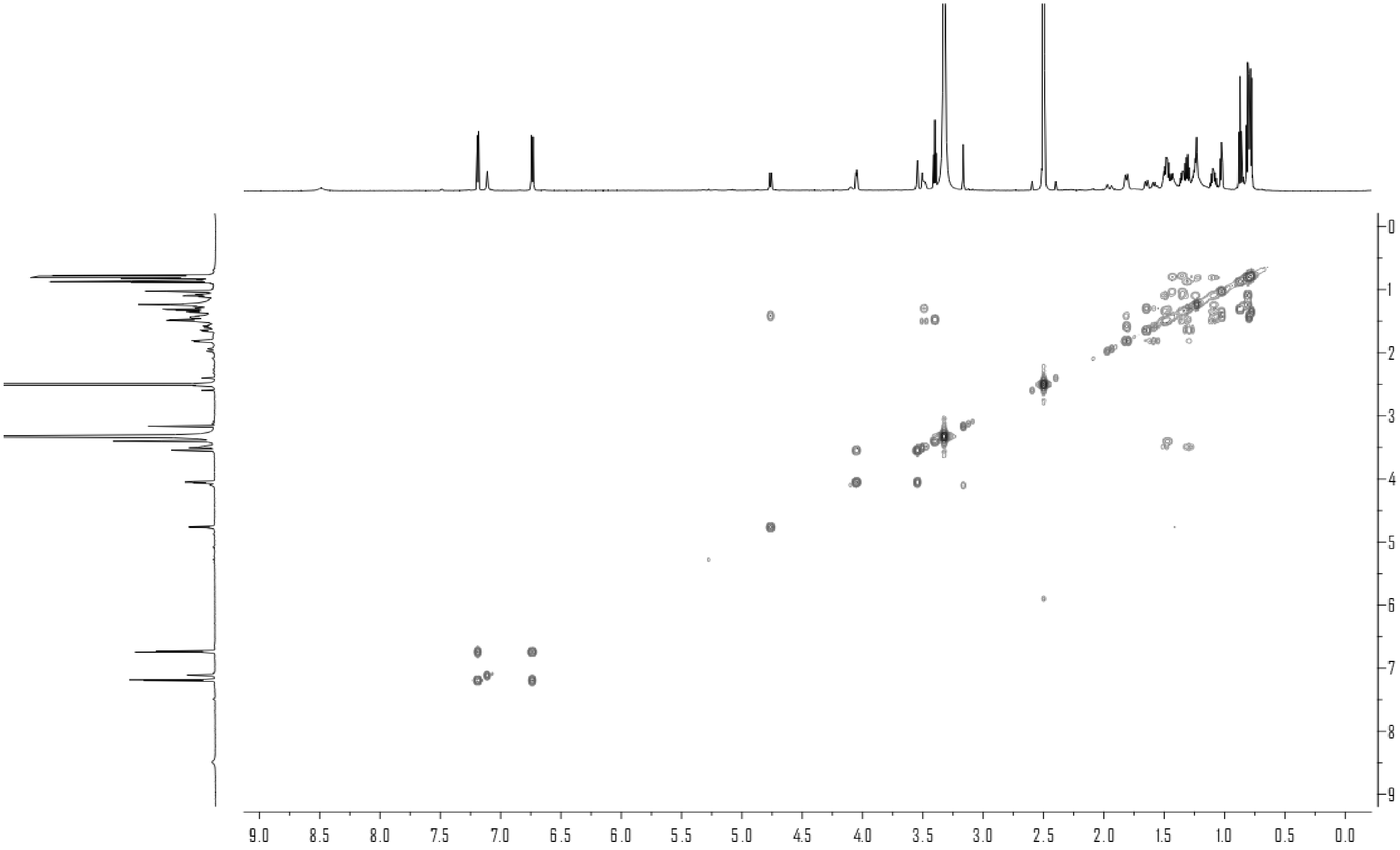

Figure S15. COSY spectrum of **3** in DMSO-d_6

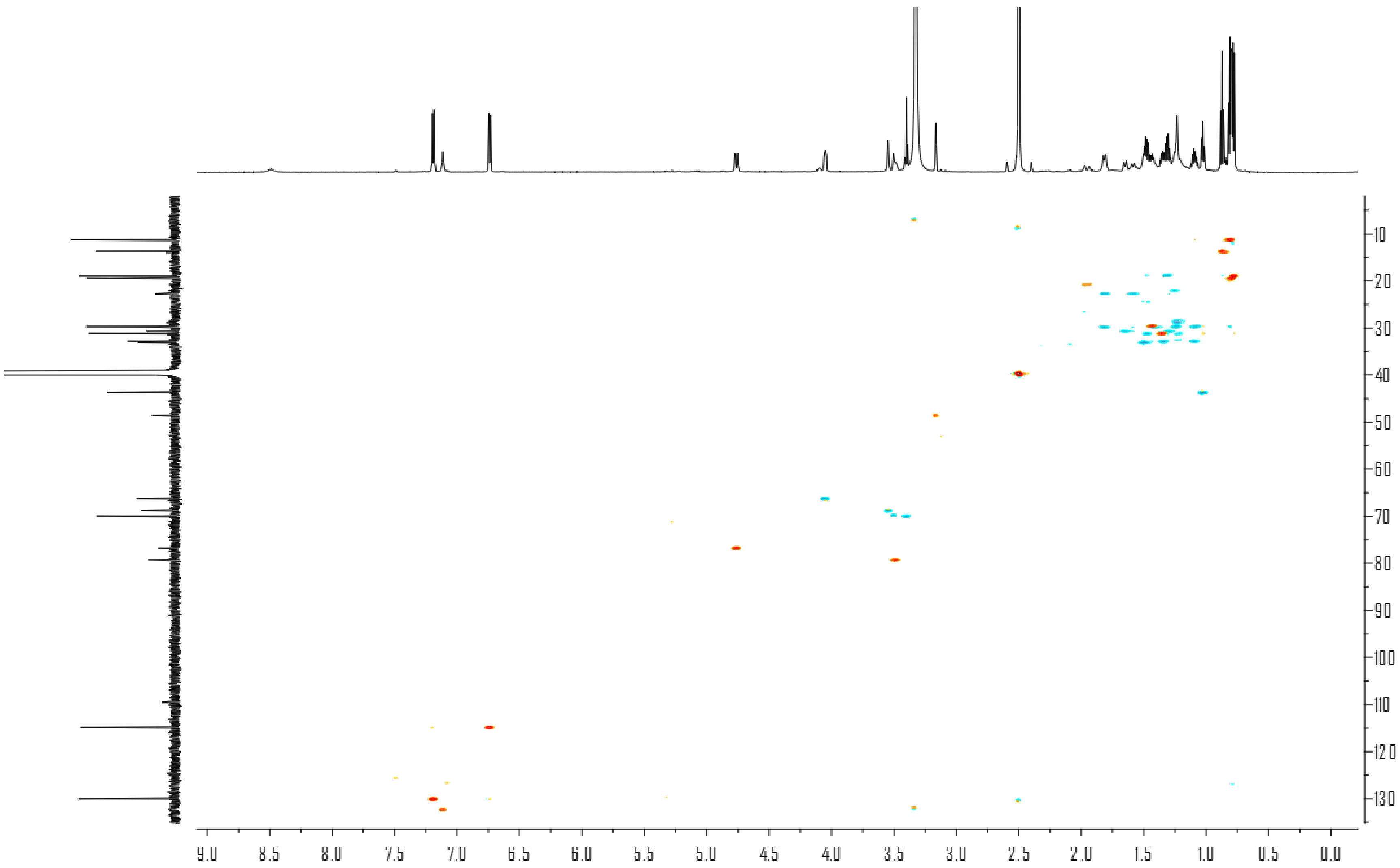

Figure S16. HSQC-DEPT spectrum of **3** in DMSO-d_6

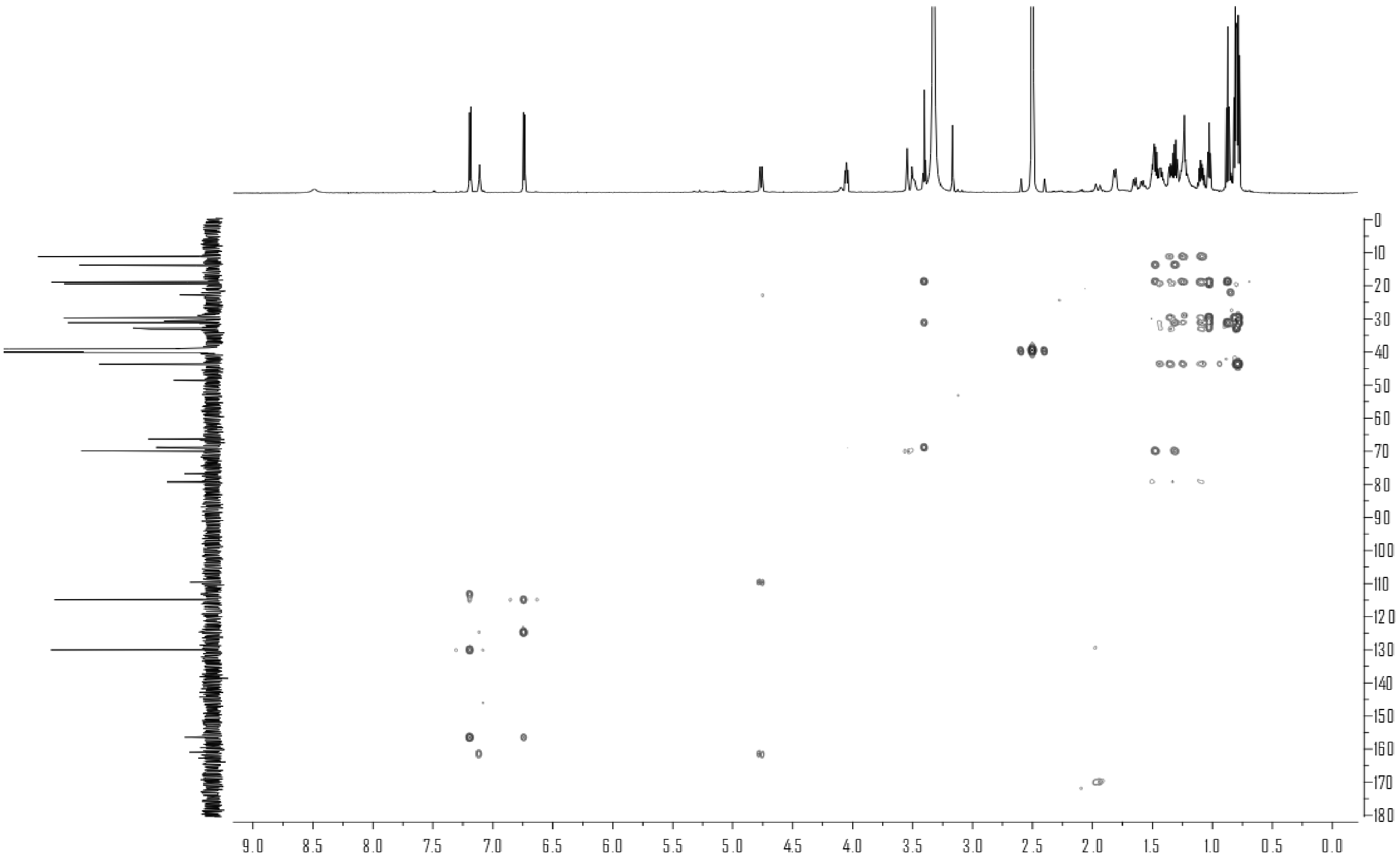

Figure S17. HMBC spectrum of **3** in DMSO-d_6

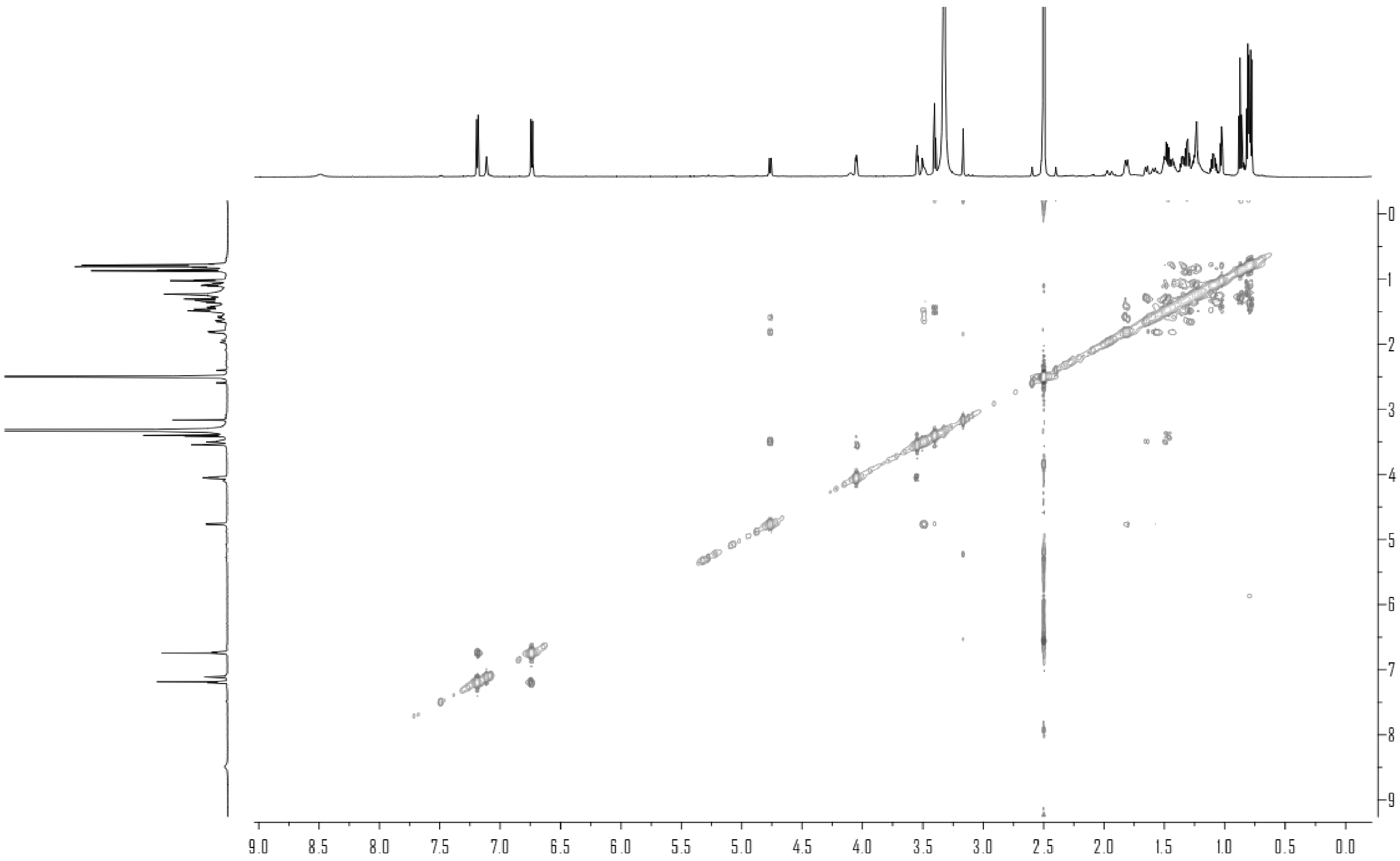

Figure S18. ROESY spectrum of **3** in DMSO-d_6

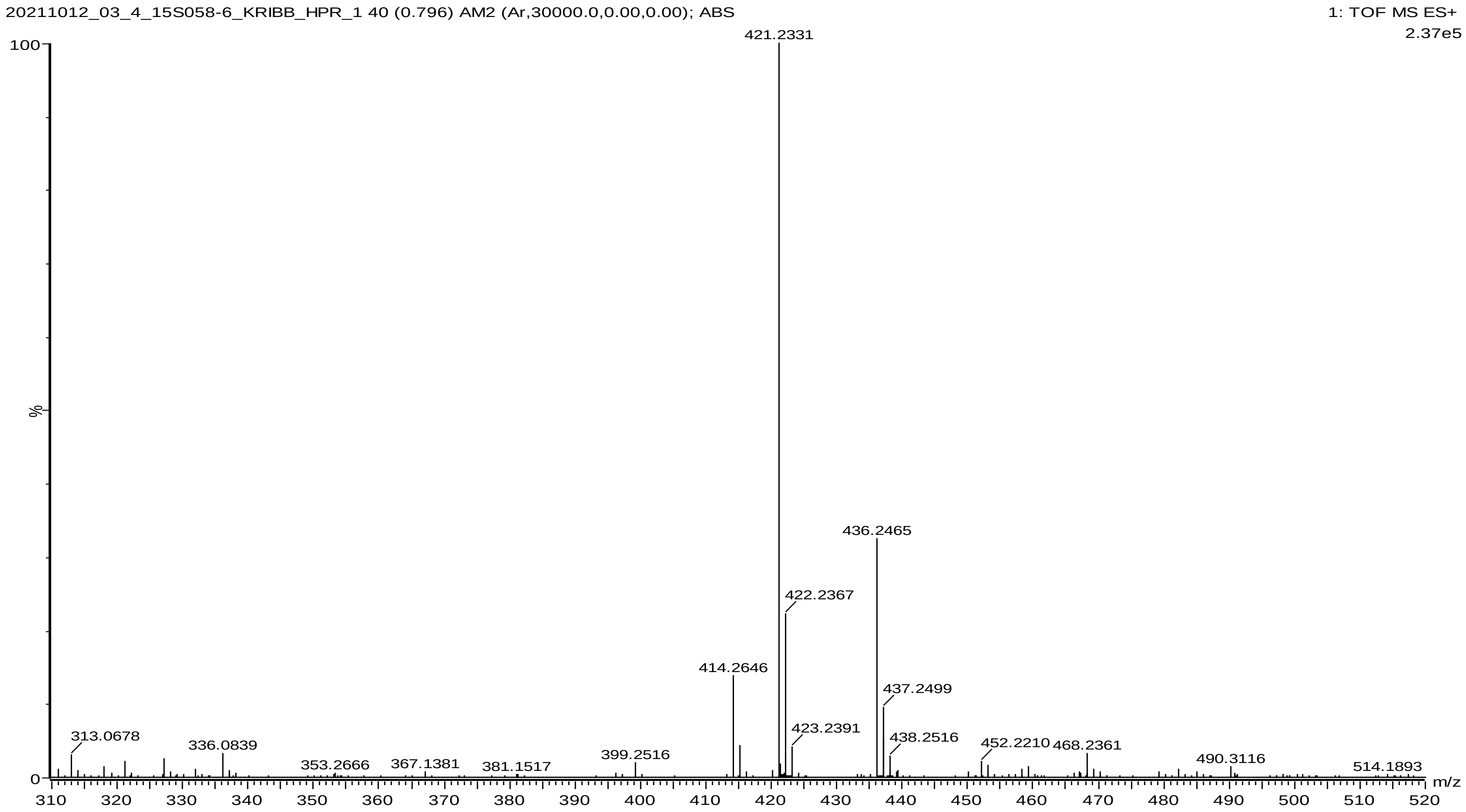

Figure S19. HRESIMS spectrum of **3**

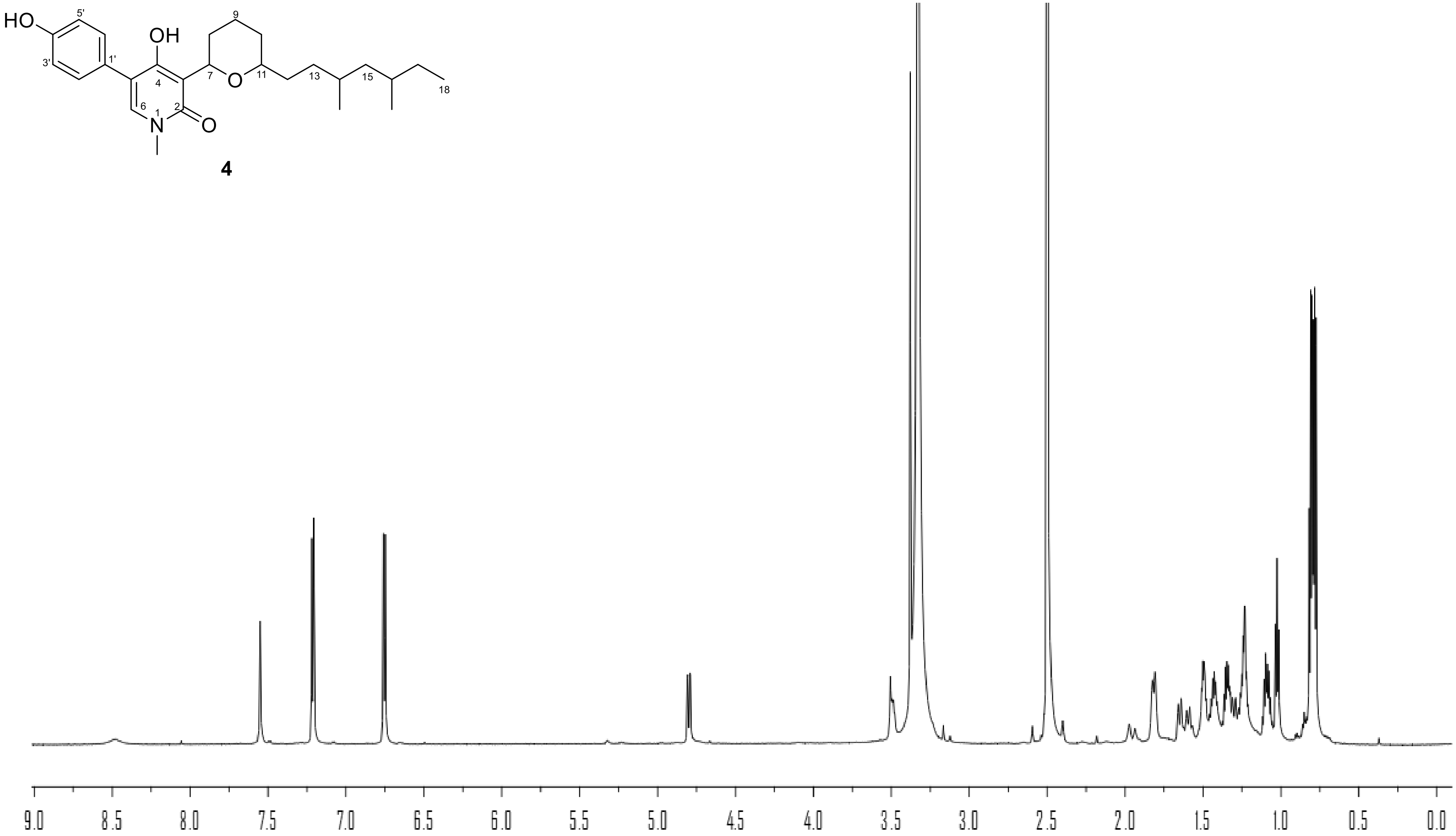

Figure S20. ^{1}H NMR spectrum (700 MHz) of **4** in DMSO-d_6

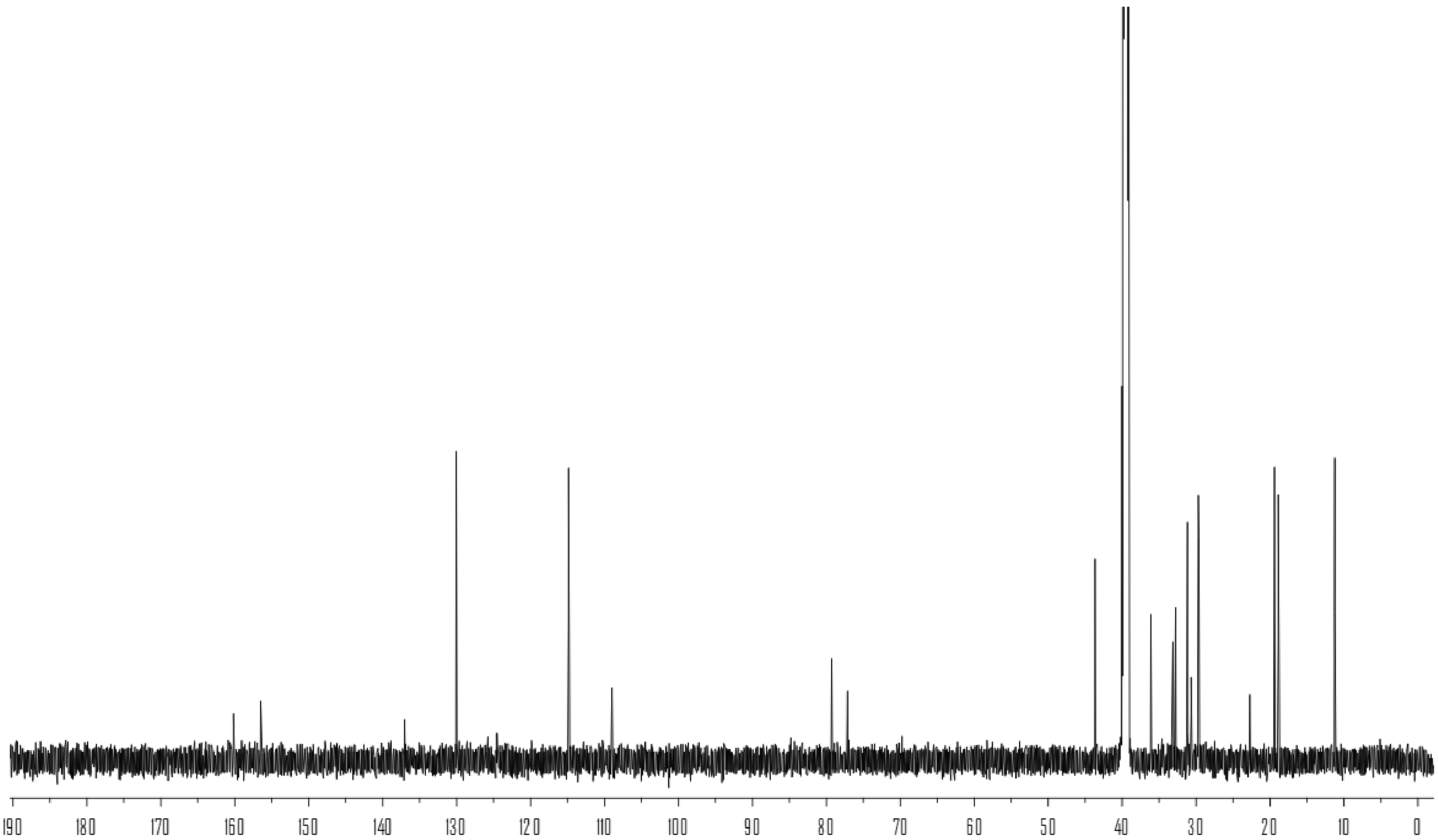

Figure S21. ^{13}C NMR spectrum (175 MHz) of **4** in DMSO-d_6

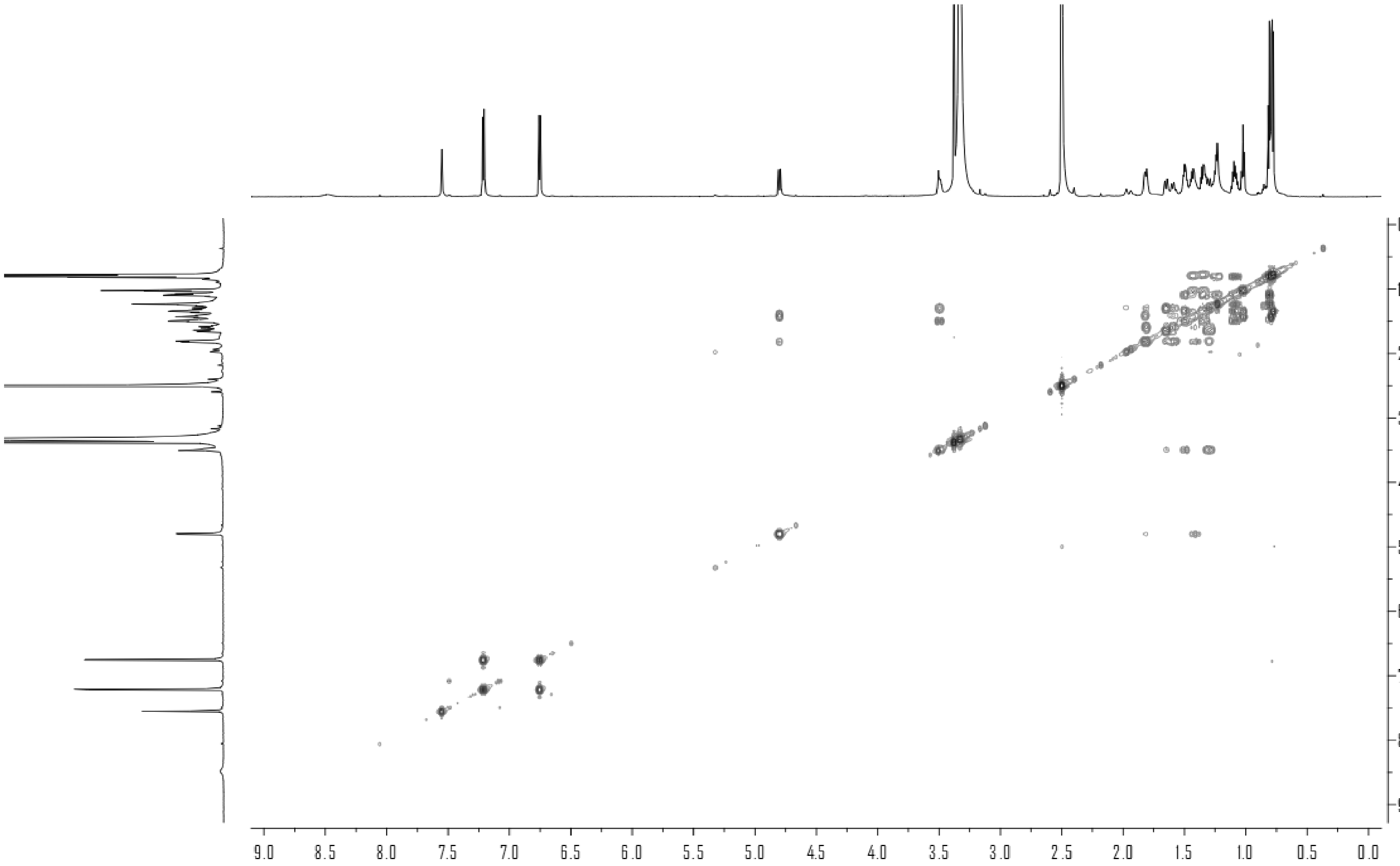

Figure S22. COSY spectrum of **4** in DMSO-d_6

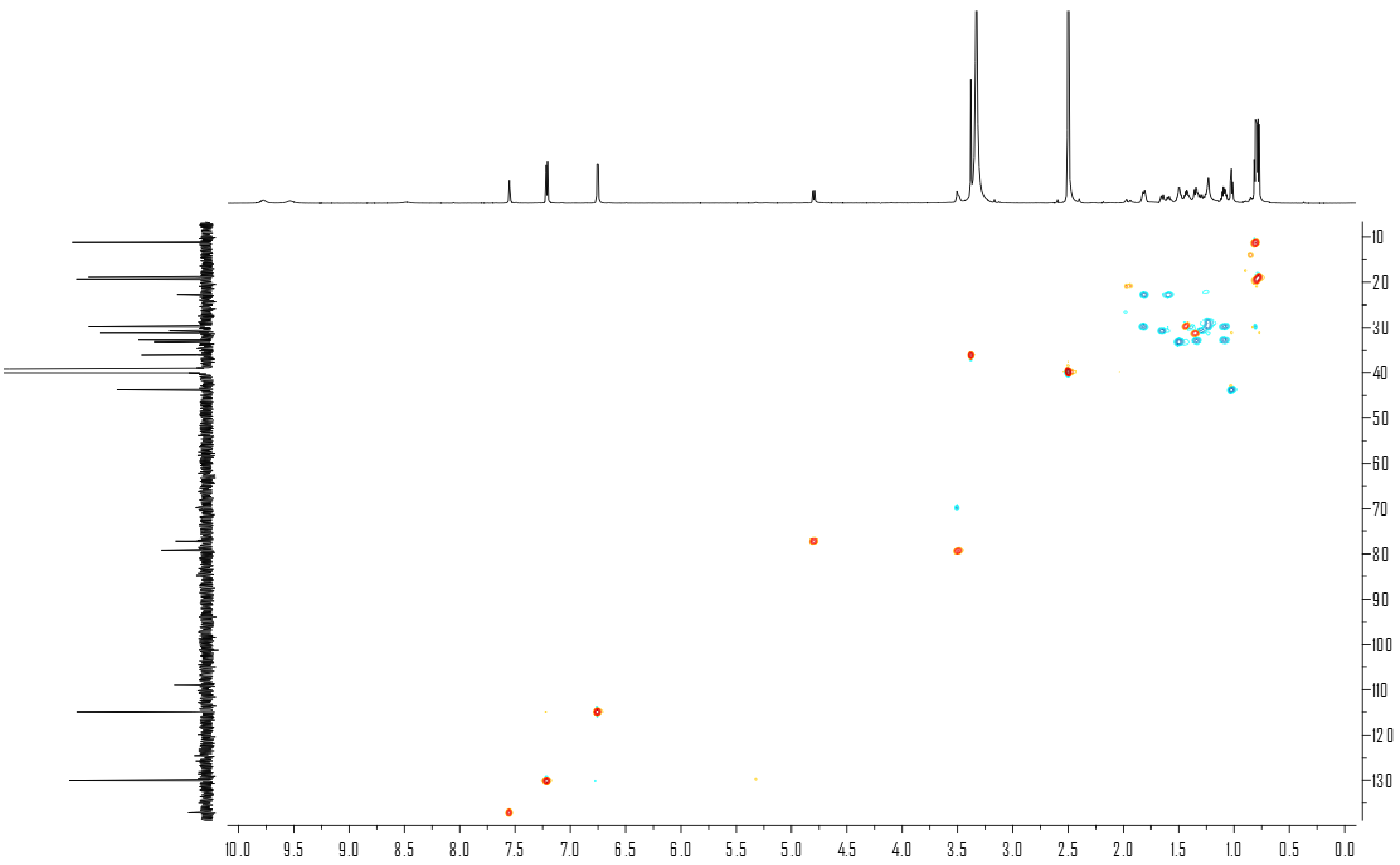

Figure S23. HSQC-DEPT spectrum of **4** in DMSO-d_6

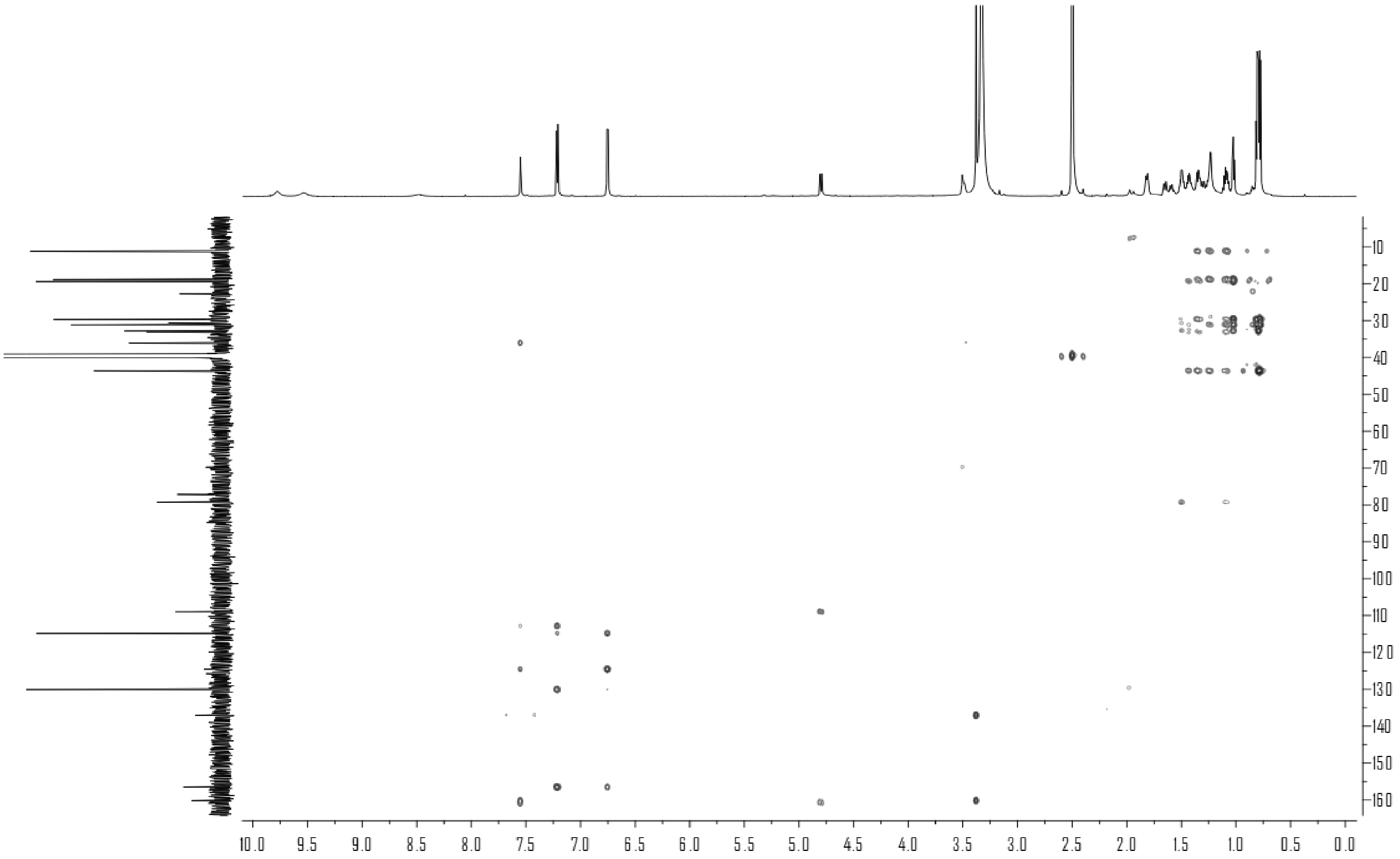

Figure S24. HMBC spectrum of **4** in DMSO-d_6

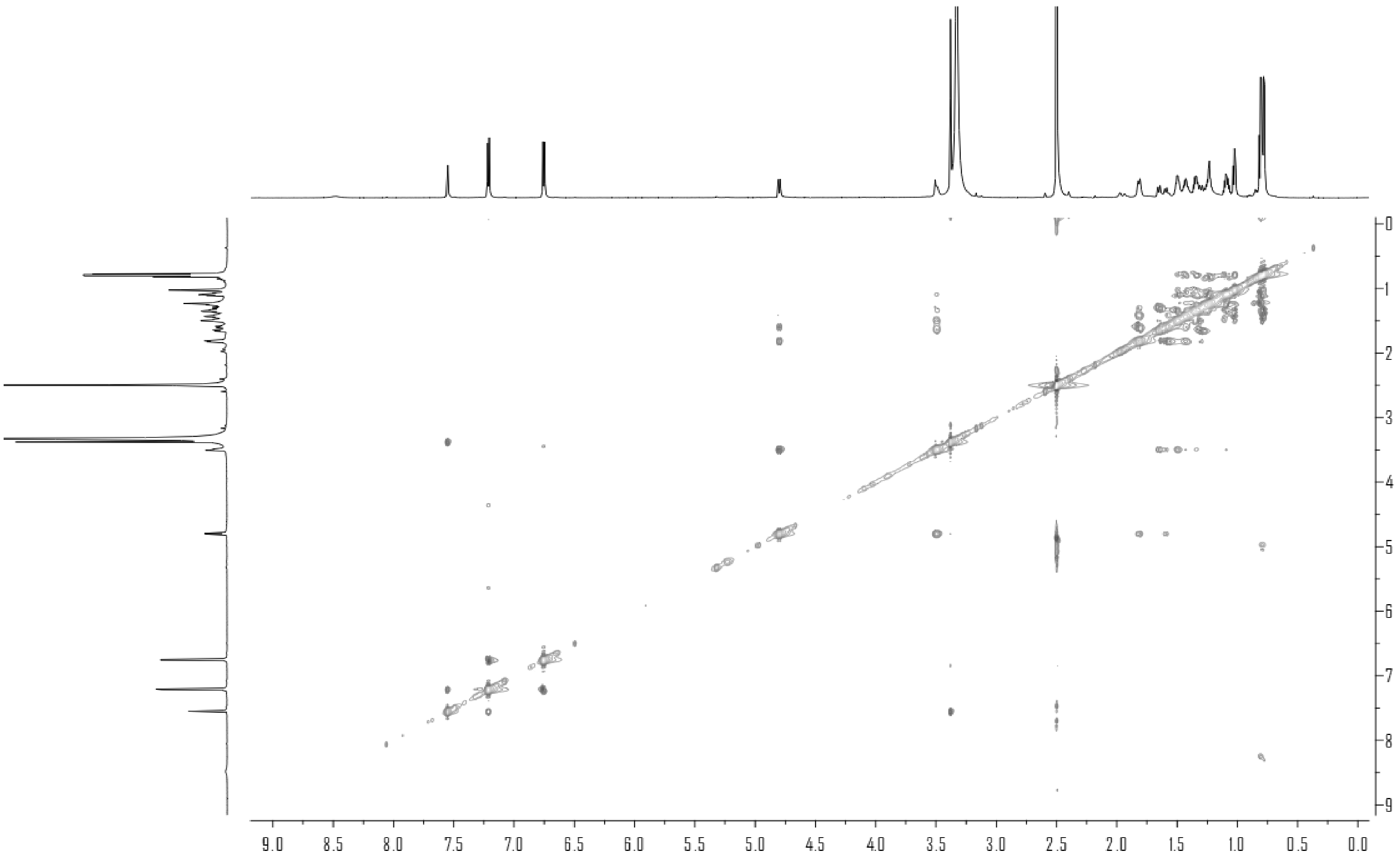

Figure S25. ROESY spectrum of **4** in DMSO-d_6

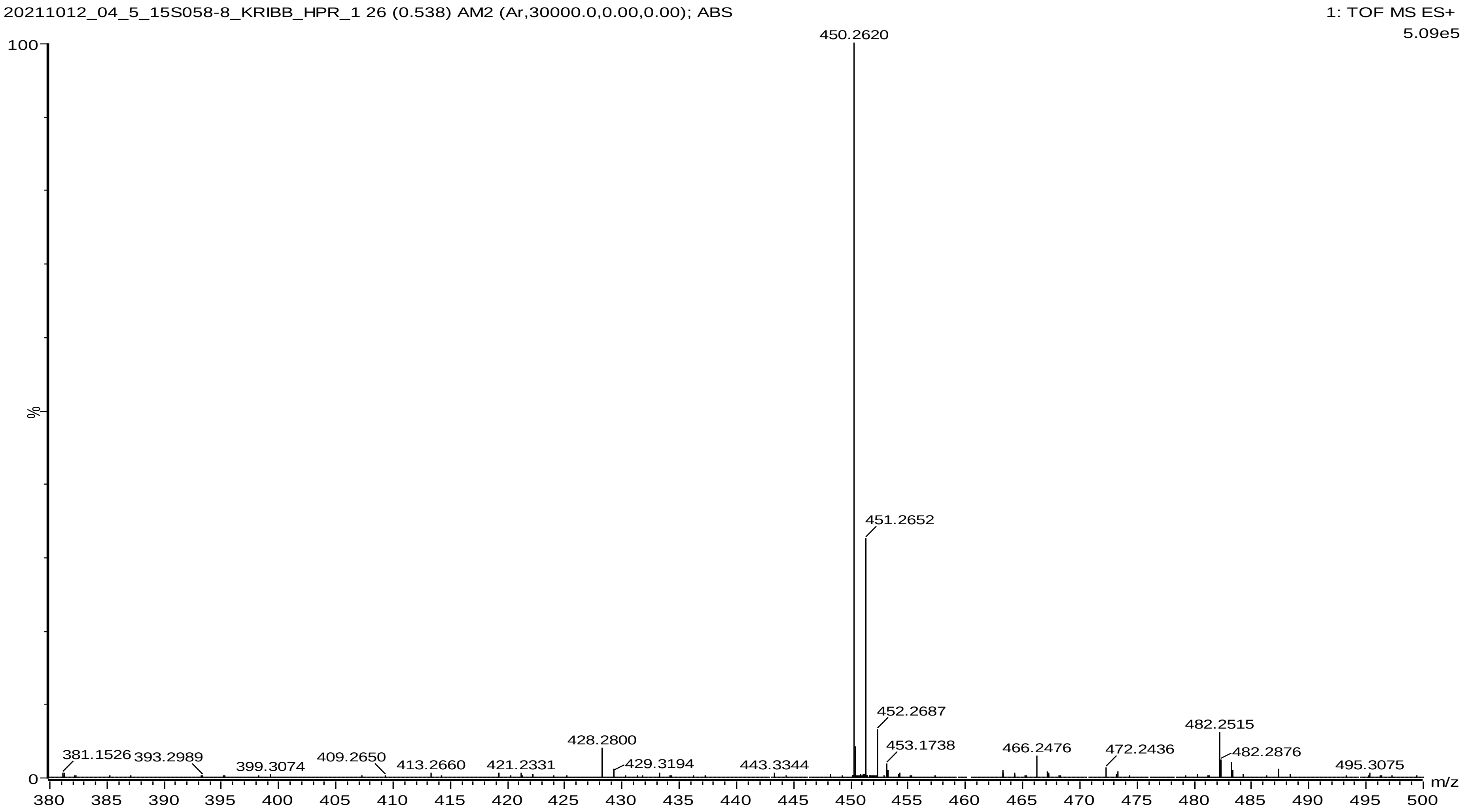

Figure S26. HRESIMS spectrum of **4**

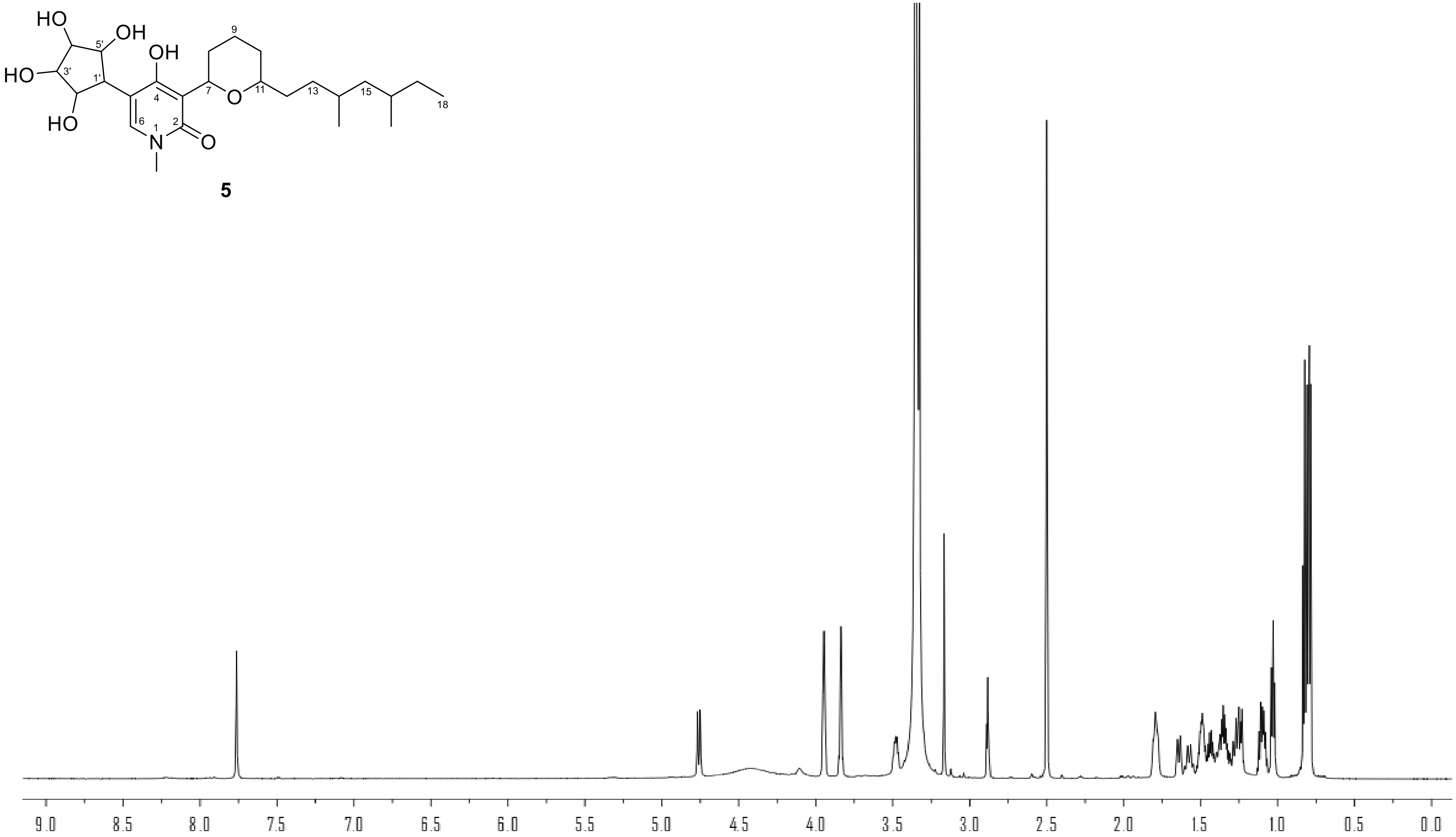

Figure S27. ^{1}H NMR spectrum (700 MHz) of **5** in DMSO-d_6

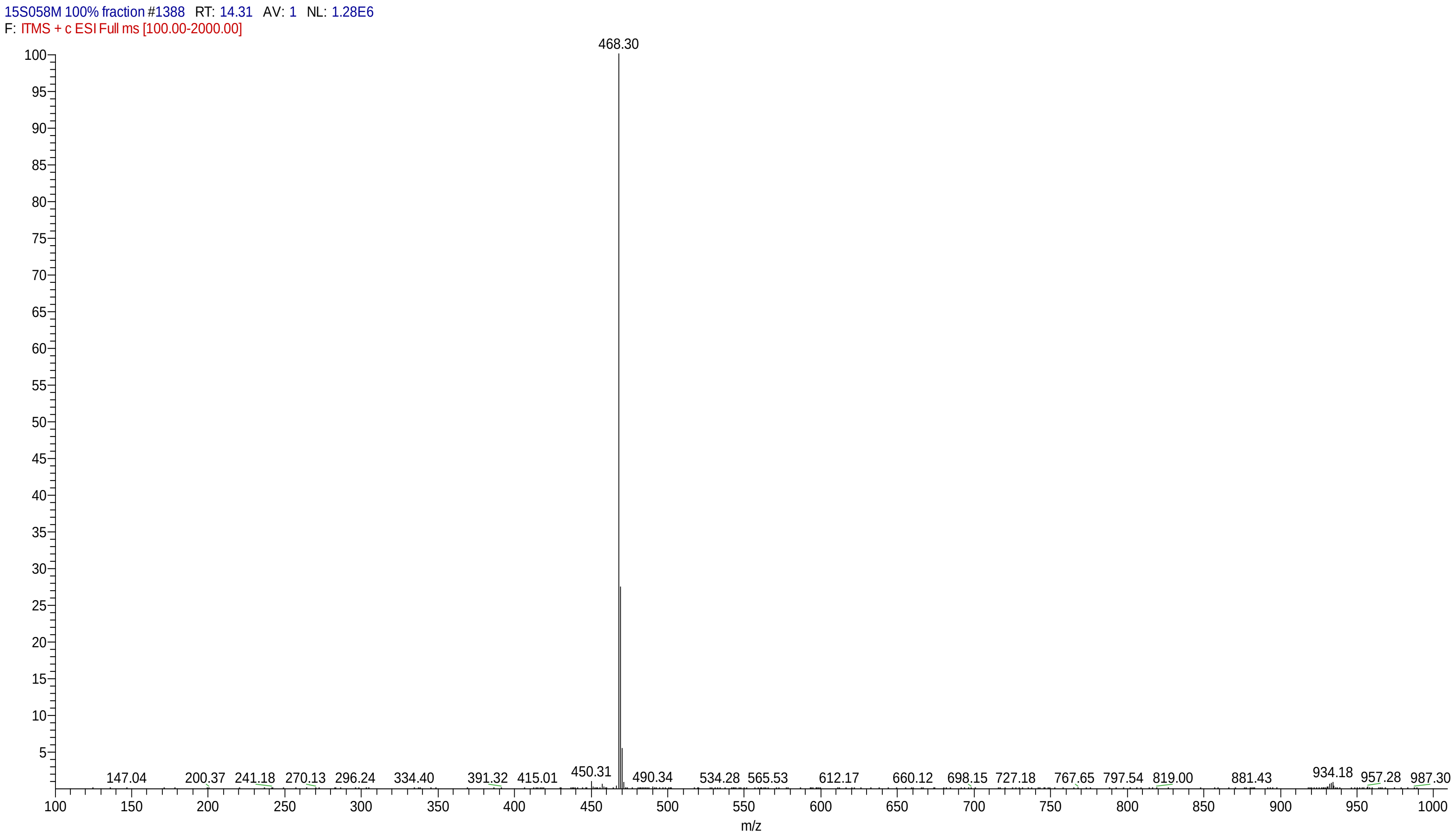

Figure S28. LRESIMS spectrum of **5**
